# Dual-branch spatio-temporal graph network for bearing fault diagnosis

**DOI:** 10.1038/s41598-026-42504-0

**Published:** 2026-03-11

**Authors:** Yajun Wang, Yang Li, Chenggang Li, Qi Dai

**Affiliations:** 1 State Energy Group Shendong Coal Group Co., Ltd., Yulin, 719315 China; 2 Shendong Technology Research Institute, CHN Energy Group, Yulin, 719315 China; 3 Branch Institute of Emergency Science, Chinese Institute of Coal Science, Beijing, 100013 China; 4 State Key Laboratory of Disaster Prevention and Ecology Protection in Open-Pit Coal Mines, Chinese Institute of Coal Science, Beijing, 100013 China; 5 College of Science, North China University of Science and Technology, Tangshan, 063210 China

**Keywords:** Rotating machinery, Fault diagnosis, Graph neural network, Dual-branch network, Engineering, Mathematics and computing

## Abstract

Bearings are one of the critical components in rotating machinery. Bearing failures can lead to equipment damage, reduced performance, and even major safety accidents. Therefore, improving the ability to diagnose bearing faults can help improve the availability, reliability, and safety of rotating machinery. However, the original vibration signals in rotating machinery often contain noise and irregularities, making it difficult for traditional vibration analysis to extract effective high-dimensional features. Inspired by the construction of spatio-temporal graphs and dual-branch graph networks, a bearing fault diagnosis method based on dual-branch spatio-temporal graph networks (DBSGN) is proposed. Firstly, the vibration signal is modeled based on spectrum theory and the spectrum analysis method to construct a spatio-temporal graph. Secondly, Laplace-based spectral decomposition is used to extract the feature vectors of samples in the spatio-temporal graph. Finally, we designed a dual-branch fusion network to train and verify the bearing data and adjusted the model’s learning of the bearing data through a dynamic attention mechanism. The experimental results on three benchmark datasets indicate that DBSGN outperforms traditional models in terms of stability and accuracy.

**Supplementary Information:**

The online version contains supplementary material available at 10.1038/s41598-026-42504-0.

## Introduction

With the rapid development of society, rotating machinery has been widely used in automation and modern industry and plays a vital role. Rolling bearings are one of the most important components in modern industrial rotating machinery. Due to long-term operation and harsh working conditions, rotating machinery such as bearings often suffers unexpected failures and structural damage. This irreversible damage may lead to economic losses such as equipment damage and reduced production capacity. In severe cases, it may even cause major safety accidents. Therefore, accurate diagnosis of rolling bearing faults has become an important research topic in the engineering field^1,2^.

At present, rolling bearing fault diagnosis technology has gone through three stages: manual diagnosis, signal processing, and artificial intelligence. Early manual diagnosis mainly relied on the sensory experience of engineering personnel, such as hearing and touch, and simple vibration measurement tools. Although intuitive, it had low diagnostic efficiency and high subjectivity, making it difficult to cope with complex working conditions and identify early subtle faults. With the advancement of signal processing technology, diagnostic methods have gradually shifted to time domain, frequency domain, and time–frequency domain analysis of vibration and acoustic emission signals. For example, technologies such as short-time Fourier transform (STFT)^3,4^, empirical wavelet transform (EWT)^5,6^, empirical mode decomposition (EMD)^7,8^, and Hilbert-Huang transform (HTT)^9^ are used to extract fault features from raw noise signals, thereby effectively identifying specific fault types. However, after discovering fault-related features, traditional signal processing methods still rely on manual or rule-based algorithms to make the final decision. In recent years, with the rise of artificial intelligence technologies such as machine learning and deep learning, rotating machinery fault diagnosis has begun to develop towards intelligence and automation. This method can learn the characteristics of different fault categories from massive monitoring data, achieve high-precision, end-to-end fault classification and status prediction, and greatly improve the accuracy and robustness of fault diagnosis.

Currently, fault diagnosis methods based on deep learning have become a research hotspot, such as convolutional neural networks (CNNs)^10,11^, recurrent neural networks (RNNs)^12,13^, graph neural networks (GNNs)^14^, and their variant models. These models autonomously extract fault features directly from input data without the need for prior knowledge, thereby performing accurate classification. When processing bearing vibration data, existing deep learning methods usually treat the data as independent time series samples or two-dimensional image data. This processing method makes it difficult to fully mine the key fault-related correlation information in the data. On the one hand, predicting continuous vibration signals as time series samples will lose the inherent correlation of bearing fault signals. On the other hand, when converting data into two-dimensional images to adapt to convolutional neural networks, although spatial features can be extracted, the fine time domain details in the original signal may be lost, and the frequency domain signal cannot be effectively modeled. These limitations result in the existing methods being insufficient and inadequate in fault feature extraction and robustness under complex operating conditions such as variable speed and strong noise, which in turn affects the stability and accuracy of the diagnosis results.

To solve the above problems, we propose a rolling bearing fault diagnosis method based on the graph neural network, namely, bearing fault diagnosis based on dual-branch spatio-temporal graph network (DBSGN). Our proposed method first models the vibration signal based on spectral graph theory and spectral analysis methods to construct a spatio-temporal graph. Secondly, Laplace-based spectral decomposition is used to extract the feature vectors of samples in the spatio-temporal graph. Finally, we designed a dual-branch fusion network to train and validate the bearing data and adjusted the model’s learning of the bearing data through a dynamic attention mechanism. The main contributions of our approach can be summarized as follows.

1. We propose a spatio-temporal graph construction method, which converts bearing vibration signals into graph-structured data, more effectively captures the complex relationships in the graph, and aggregates complex information in neighbor nodes through a message passing mechanism.
2. We propose a bearing fault detection model based on a dual-branch fusion network, which enhances the accuracy and robustness of the model and stabilizes the detection results.
3. This study conducts experimental verification on three widely used public datasets, and the experimental results demonstrate the effectiveness of our method in distinguishing abnormal signals from normal signals and achieving more efficient and accurate fault identification.

The rest of this paper is structured as follows. Section “Related work” introduces graph neural networks and a review of related literature. Section “Preliminaries” gives the experimental preparation and the theoretical basis of graph convolution. Section “The dual-branch space–time graph network” describes the proposed fault detection model in detail. In Sect. “Experiment”, we conduct experiments and conduct an in-depth analysis of the experimental results to verify the effectiveness of the model. In Sect. “Conclusion and future work” we conclude our work and discuss future directions.

## Related work

### Machine learning

With the rapid development of machine learning, more and more researchers have begun to use machine learning to predict bearing fault signals. Common machine learning methods include random forest (RF), principal component analysis (PCA), K nearest neighbor (KNN), support vector machine (SVM), etc. Applying these methods requires deep exploratory data analysis of the dataset and feature engineering to extract features from vibration signals. Finally, it is passed to the ML algorithm for prediction. Chen et al.^15^ proposed an imbalanced sample diagnosis framework based on clustering and density methods, which improved performance and interpretability by adjusting sample distribution and fusing multi-domain features. Transfer learning methods have also been applied across different working conditions and machines. Wan et al.^16^ proposed a cross-domain adaptation network that combines layered decoding and an attention mechanism, enabling cross-machine diagnosis without labels. Cui et al.^17^ combined unsupervised learning and sensitivity analysis in their research on wind turbines and proposed a three-stage learning framework to effectively extract features under diverse working conditions. Wang et al.^18^ proposed an application method that combines numerical simulation models with machine learning classification. They used a mature dynamic model to build a simulation model, modified the fault influencing factors through the Pearson correlation coefficient (PCC), obtained simulation data, and used the machine learning model for prediction. Habbouche et al.^19^ proposed an early diagnosis method for bearing faults based on variational mode decomposition (VMD) and a machine learning method, which uses VMD for feature extraction for fault detection and then triggers the use of a one-dimensional convolutional neural network for multi-scale feature extraction for classification and diagnosis. Brusamarello et al.^20^ proposed a support vector machine (SVM) classifier to identify the fault severity level by selecting the four highest peaks in the spectrum and PCA techniques for feature extraction, and used a supervised SVM classifier for prediction.

### Deep learning

Although machine learning methods have achieved good performance in bearing fault detection, there are still some challenges, such as the need for extensive domain expertise, complex feature engineering, and data explosion. In order to solve the above problems, more and more researchers have adopted deep learning algorithms to perform fault diagnosis on bearing vibration signals and have achieved good model performance and experimental results. Common deep learning algorithms include convolutional neural networks (CNN), autoencoders, long short-term memory networks (LSTM), generative adversarial networks (GAN), etc. Compared with classic ML algorithms, these DL algorithms have the advantages of automatic feature extraction and good transferability. Hoang D et al.^21^ systematically reviewed the application of deep learning methods such as autoencoders, restricted Boltzmann machines and convolutional neural networks in bearing diagnosis, pointing out their advantages in automatic feature learning. Neupane and Seok^22^ specifically summarized the current status of deep learning research based on the CWRU dataset, explaining that this dataset has become an important benchmark for validating diagnostic methods. In terms of specific methods, Hatipoğlu et al.^23^ proposed a framework that combines multi-domain features, LSTM and attention mechanism, and achieved high-precision diagnosis on both CWRU and HUST datasets. Rezazadeh et al.^24^ proposed an unsupervised domain adaptation framework called WaveCORAL-DCCA, which innovatively combines discrete wavelet transform for robust time–frequency feature extraction with an enhanced deep canonical correlation analysis (DCCA) network and incorporates a correlation alignment (CORAL) loss function to achieve superior domain-invariant representation learning. Du et al.^25^ proposed VMD decomposition combined with GASF-CNN and BiLSTM parallel network to improve the performance under complex working conditions. To address the sample imbalance problem, Li et al.^26^ proposed a diagnostic method combining CNN-KAN with diffusion network enhancement, which significantly improved the accuracy under few-sample conditions. Ma and Guo^27^ combined time–frequency joint features with a hybrid deep network to achieve robust diagnosis in variable speed and strong noise environments. Xie et al.^28^ combined the enhanced VAE-WGAN with the WDCNN dual-channel structure, showing superior performance under class imbalance conditions. In addition, scholars at home and abroad have proposed a variety of improvement strategies, such as ACGAN-SN based on spectral normalization^29^, capsule GAN combined with wavelet features^30^, deep feature enhancement GAN^31^, etc., which have made significant progress in the diversity and reliability of generated samples.

### Graph neural networks

Although rolling bearing data contains rich relational structures, practical engineering often faces the problem of limited sample size, which makes the model unable to fully explore the features in the bearing data. To address this problem, graph neural networks have become an effective method in the field of rolling bearing fault diagnosis in recent years due to their ability to model structured data. Yang et al.^32^ proposed a super graph method to convert the original vibration signal into graph structured data, and then used a graph convolutional network for final diagnosis. Yu et al.^33^ proposed a new fault diagnosis framework based on graph neural network, which converts the features extracted from the original vibration signal into a graph structure and applies GNN to the graph data for fault diagnosis research. Zhang et al.^34^ constructed an adjacency matrix through feature extraction and causal test quantization weights and input it into GNN classification. This method effectively alleviated the impact of noise on vibration signals and enhanced the interpretability of the model. Jiang et al.^35^ proposed a multi-head graph attention network, which aggregates the discriminative features of graph data into new feature representations through a multi-head attention mechanism. Sun et al.^36^ introduced a novel intelligent fault diagnosis method based on frequency-time sequence graph (FTSG) and graph generative classification adversarial network. Certain frequencies in the spectrum are used as node features, connected by edges in chronological order, and the generator is used to expand the training samples, effectively alleviating the impact of the scarcity of fault samples. Xiao et al.^37^ simplified the generated weighted graph structure data by defining an edge failure threshold to improve data quality and improved the graph convolutional network (GCN) to reduce computational complexity. Rezazadeh et al.^38^ proposed a novel structural health monitoring (SHM) framework for composite materials under temperature-induced variations. This method leverages the powerful feature extraction capabilities of GAT and advanced domain adaptation (DA) techniques, achieving scalable comparison of temperature domain feature distributions by combining maximum mean discrepancy (MMD) and correlation alignment (CORAL) losses with a domain discriminative adversarial model.

## Preliminaries

### Problem definition

A graph $G$ is represented as $G = (V,E,X,A)$, where $V = \{ v_{i} |i = 1,2,...,n\}$ represents the set of nodes in the graph $G$, $E = \{ e_{i} |i = 1,2,...,m\} \subseteq V \times V$ represents the set of connected edges in the graph $G$, $X \in {\mathrm{R}}^{n \times d}$ represents the node features, and $A \in \{ 0,1\}^{n \times n}$ represents the adjacency matrix, $|V| = n$ and $d$ are the number of nodes and the feature dimension of each node, respectively. $A = [a_{ij} ]$ represents the relationship between nodes $v_{i}$ and $v_{j}$. If there is an edge between $v_{i}$ and ${\mathrm{v}}_{{\mathrm{j}}}$ (i.e., $(v_{i} ,v_{j} ) \in {\mathrm{E}}$), then $a_{ij} = 1$, otherwise $a_{ij} = 0$. In addition, the labels of fault classes are denoted as $C = \{ c_{i} |i = 1,2, \ldots ,C\}$, where $c_{i}$ represents the label belonging to a specific class and $C$ represents the total number of classes.

### Theoretical basis

Graph convolutional networks (GCNs)^39^ represent a class of neural networks specifically designed to process graph-structured data. They can be roughly divided into spectral methods rooted in graph signal processing and spatial methods that directly define convolutions on graph topology. The GCNs used in this work belong to the spectral domain category. However, traditional spectral graph convolution has two limitations. First, the graph convolution kernel is global and has a large number of parameters. The size of the convolution kernel is the same as the input signal, and the number of parameters is the same as the number of graph nodes. Second, the graph convolution operation is highly complex and relies on the inverse operation of the adjacency matrix. The computational complexity is $O({N}^{2})$. When $N$ is large, the amount of calculation is too large, which is not conducive to deep network training. In order to solve the above problems, Chebyshev proposed to use polynomial expansion to approximate the calculation of graph convolution^40^, that is, to perform polynomial approximation on the parameterized frequency response function:

1 $$\begin{array}{*{20}c} {g_{\theta } \left( \Lambda \right) \approx \sum\limits_{k = 0}^{K - 1} {\theta_{k} \Lambda^{k} } } \\ \end{array}$$

Where $k$ represents the highest order of the polynomial. The number of parameters of the convolution kernel is reduced from $n$ to $k$, but the complexity of the entire graph convolution operation is still $O\left( {N^{2} } \right)$.

To further reduce computational complexity, Defferrard et al.^41^ used the $k$-th order Chebyshev polynomial $T_{k} (x)$ to better approximate traditional graph convolution and proved that the complexity can be reduced to $O\left( {N|E|} \right)$. The equation definition is as follows:

Where $g_{\theta }{\prime} ( \cdot )$ represents the filter function, $\theta_{k}$ is the trainable coefficient of the Chebyshev coefficient, and $T_{k} (x)$ is the recursive calculation of the Chebyshev polynomial, as shown in Eq. 2. $\tilde{\Lambda }$ is the normalized version of $\Lambda$, and the calculation formula is shown in Eq. 3:

2 $$\left\{ \begin{aligned} T_{0} \left( x \right) = & 1,\;T_{1} \left( x \right) = x \\ T_{k} \left( x \right) = & 2xT_{k - 1} \left( x \right) - T_{k - 2} \left( x \right),\;k \ge 2 \\ \end{aligned} \right.$$

3 $$\begin{array}{*{20}c} {\tilde{\Lambda } = \frac{2\Lambda }{{\lambda_{\max } }} - I_{N} } \\ \end{array}$$

Where ${\lambda }_{max}$ represents the maximum eigenvalue of the diagonal matrix ${\Lambda }$, and the eigenvalues in ${\tilde{\Lambda }}$ range from $\left[ { - 1,1} \right]$.

By multiplying the Chebyshev graph convolution $g_{\theta }{\prime}$ with the feature matrix $X$ of the input signal, the aggregation of multi-order neighbor features is achieved, and the feature matrix after dimensionality reduction is finally output. The calculation formula for Chebyshev graph convolution feature aggregation is shown in Eq. 4:

4 $$\begin{array}{*{20}c} {M = \sum\limits_{k = 0}^{K - 1} {\theta_{k} UT_{k} \left( {\tilde{\Lambda }} \right)U^{T} X} } \\ \end{array}$$

The graph convolution operation can be viewed as filtering the signal without changing the feature dimension. After the graph signal is multiplied by the trainable weight matrix, the dimension of the feature can be changed, as shown in Eq. 5:

5 $$\begin{array}{*{20}c} {X^{i} = Cheb\left( {M, W^{i} } \right) = MW^{i} } \\ \end{array}$$

Where ${\mathrm{W}}^{{\mathrm{i}}}$ is the trainable weight matrix of the $i$-th graph convolution layer, $Cheb\left( { \cdot , \cdot } \right)$ represents the function of Chebyshev convolution, and $X^{i}$ is the final output of the $i$-th graph convolution layer.

Referring to previous work^42,43^, this experiment selected five Chebyshev convolutional layers as the prediction model. The definition of its prediction model is shown in Eq. 6:

6 $$\begin{array}{*{20}c} {Y = softmax \left( {Cheb\left( {\sigma \left( {Cheb\left( {X^{i} ,W^{i} } \right)} \right)} \right)} \right)} \\ \end{array}$$

Where $softmax ( \cdot )$ is the normalized exponential function, and $\sigma ( \cdot )$ represents the activation function rectified linear unit (RELU).

## The dual-branch space–time graph network

**Fig. 1:**
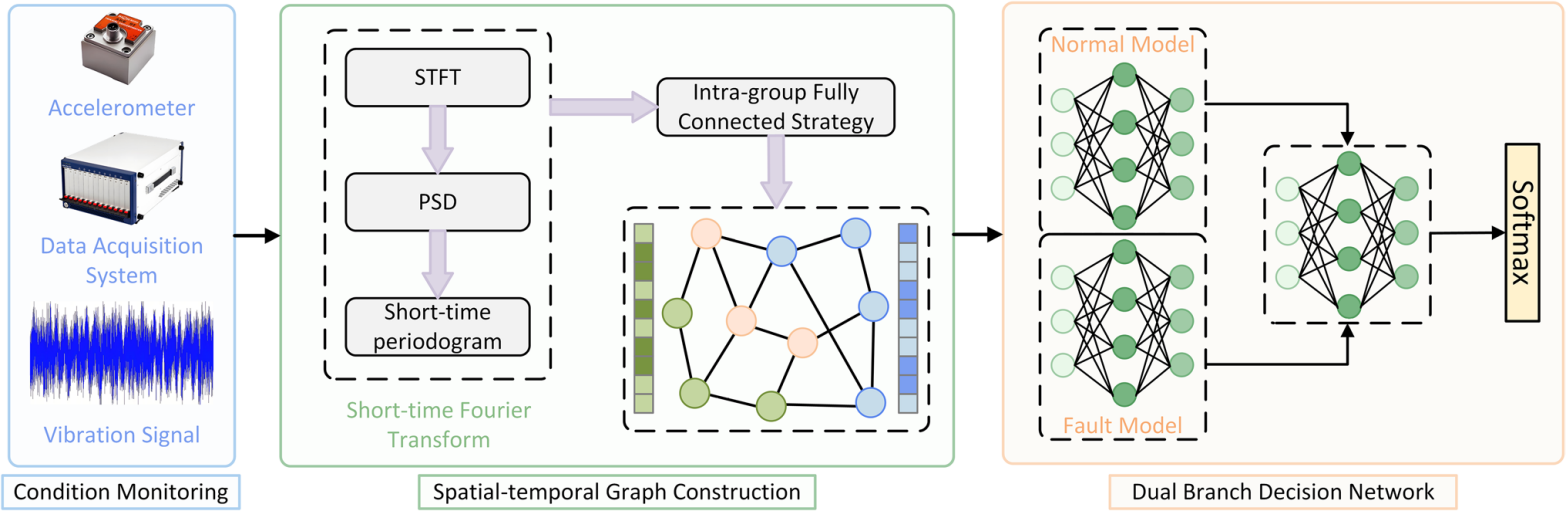
Schematic diagram of a new model for fault diagnosis of rolling bearings based on DBSGN.

This paper introduces in detail the proposed new rolling bearing fault diagnosis model based on dual-branch spatio-temporal graphs network (DBSGN) and derives the calculation process of the model. The overall architecture of DBSGN is shown in Fig. 1. The model is mainly divided into three parts: spatio-temporal graph generation, dual-branch decision network and decision fusion. In Sect. “Spatial–temporal graph generation”, the process of generating a spatio-temporal graphs from the rolling bearing dataset is described in detail. In Sect. “Dual-branch decision network”, a dual-branch Chebyshev network is used to learn the characteristic patterns of different data in the bearing fault dataset. Finally, the final decision is generated through an adaptive decision mechanism to improve the robustness of the final classification decision, as shown in Sect. “Decision fusion”.

### Spatial–temporal graph generation

In bearing fault diagnosis, traditional deep learning models perform bearing fault diagnosis by constructing time–frequency domain features into time series samples or two-dimensional image data. These methods are difficult to simultaneously capture the temporal dynamics, spatial correlation, and frequency distribution characteristics of the fault, resulting in weak prediction accuracy and robustness of the model. To address this issue, we propose a spatio-temporal graph generation framework to convert unstructured bearing vibration signals into graph-structured data, thereby capturing the spatial correlation and temporal dynamics between features and providing an adaptive input format for graph convolutional networks.

The original bearing vibration signal contains a lot of noise, and it is impossible to extract effective fault features. In order to preserve the local time domain characteristics of the fault, this paper adopts a non-overlapping sliding window strategy to segment the input vibration signal and provide standardized input for subsequent time–frequency analysis.

Since the generated normal and faulty samples have high similarity in time domain waveforms, it is difficult to accurately distinguish normal samples from faulty samples only through the time domain waveforms. In order to further improve the recognition of fault features, this paper uses short-time Fourier transform (STFT) to map time domain samples to the time–frequency domain, present the fault signal features in three-dimensional form, and improve the visibility of fault features. The STFT calculation formula in the continuous time–frequency domain is shown in Eq. 7:

7 $$\begin{array}{*{20}c} {S\left( {\tau ,f} \right) = \mathop \smallint \limits_{ - \infty }^{ + \infty } x\left( t \right) \cdot \omega \left( {t - \tau } \right) \cdot e^{ - j2\pi ft} dt} \\ \end{array}$$

Where $S(\tau ,f)$ is a complex time–frequency matrix, $f$ is the frequency parameter, $\tau$ is the time frame parameter, $x\left( t \right)$ represents the time domain signal of the short-time sample, $t$ represents the range of the sliding window length, and $\omega \left( {t - \tau } \right)$ represents the selected window function. The choice of window function directly affects the time–frequency analysis effect and needs to meet the requirements of high sidelobe attenuation rate and moderate mainlobe width. This study uses Hanning window as the sliding window, and its formula is expressed as:

8 $$\begin{array}{*{20}c} {\omega \left( t \right) = 0.5\left[ {1 - \cos \left( {\frac{2\pi t}{T}} \right)} \right]} \\ \end{array}$$

Where $Ts$ represents the window length of the STFT.

Through short-time Fourier transform, the transformation of time domain signals into time–frequency features is achieved, preserving the temporal dynamics and frequency distribution characteristics of the fault. However, the amplitude of the time–frequency matrix output by the short-time Fourier transform can only reflect the signal intensity and cannot directly quantify the energy distribution. To solve the above problems, the power spectral density (PSD) is used to convert the amplitude of the time–frequency matrix into an energy index with clear physical meaning, amplifying the energy difference between the fault characteristics and the normal characteristics, making the energy peak at the fault characteristic frequency more significant. The calculation formula for PSD is shown in Eq. 9:

9 $$\begin{array}{*{20}c} {PSD\left( {s,f,t} \right) = \frac{{\left| {S_{s,f,t} } \right|^{2} }}{T}} \\ \end{array}$$

Where $PSD\left( {s,f,t} \right)$ represents the power spectral density value, $S_{s,f,t}$ represents the short-time Fourier transform assignment matrix, and $T$ represents the Hanning window length.

Traditional deep learning models are usually used to process image data or time series data. Traditional deep learning models are typically used to process image data or time series data. In contrast, GNN models use the adjacency matrix $A$ and feature matrix $X$ in the graph data $G$ as input, fully utilizing the hidden structural and topological information in the graph to improve the accuracy of model predictions. However, the process of how to convert one-dimensional vibration signal data into Euclidean data deserves careful consideration. To this end, this paper proposes a spatio-temporal graph construction method based on the Laplace matrix to solve the above problems. Compared with traditional feature extraction methods, spatio-temporal graphs establish a mapping relationship between time–frequency features and graph structure, so that the graph not only includes time–frequency domain information, but also contains the structural information and topological relationship of the graph, further improving the discrimination of the extracted features and the prediction accuracy of the GNN model. The specific construction process of the spatial–temporal graph $G$ is as follows:

After processing the vibration signals in the bearing dataset using STFT, we treat all the resulting fault samples as nodes in graph $G$. Subsequently, we group all the nodes $V$ and construct the graph. Due to the spatio-temporal-frequency global coupling characteristics of bearing vibration signals, fault features often exist in the form of discrete frequency bands or their harmonic clusters, and the coupling relationships are complex. A fully connected topology within each group can maximize the preservation of potential correlation information within the group and prevent the loss of weak fault features. Therefore, we use a fully connected strategy within each group to construct the spatio-temporal graph $G$ for all nodes in different groups.

In order to obtain the features of each node in the graph, we first define a weight matrix $W$. This paper uses Euclidean distance and Gaussian kernel to calculate the power spectral density matrix of each node to measure the similarity value between different frequencies of each node. The greater the feature similarity between different frequencies, the higher the weight value in the adjacency weight matrix, and the better the effect of subsequent feature extraction. The calculation formula of the weight matrix $W$ is shown in Eq. 10:

10 $$\begin{array}{*{20}c} {W_{ij} = \exp \left( { - \frac{1}{{\sigma^{2} }}\mathop \sum \limits_{{t = t_{0} }}^{{t_{m} }} DisPSD\left( {F_{i} ,t} \right),PSD\left( {F_{j} ,t} \right)} \right)} \\ \end{array}$$

Where $W_{ij}$ represents the elements of the undirected adjacency weight matrix, with a value range of ${(0,1]}$. The weight of nodes with exactly the same features is 1, and the weight of nodes with very different features tends to 0. $i$ and $j$ are node indices of different frequencies. $PSD(F_{i} ,t)$ represents the power spectral density value of the $F_{i}$-th frequency at a certain time point $t$. $Dis\{ \cdot , \cdot \}$ represents the Euclidean distance between frequencies $F_{i}$ and $F_{j}$ at a certain time point $t$, which is a commonly used distance function. $\sigma$ is the Gaussian kernel parameter used to control the degree of influence of distance on weight.

In spectral graph analysis, the symmetric normalized Laplacian matrix is the core input of graph convolution, which updates the current node features through the features of neighboring nodes to avoid the interference of isolated nodes on feature learning. Therefore, we use the symmetric normalized Laplace matrix to process the weight matrix. First, calculate the degree matrix $D$ of the weight matrix. The calculation formula of the Laplace matrix $L$ is shown in Eqs. 11–12:

11 $$\begin{array}{*{20}c} {L = D - W} \\ \end{array}$$

12 $$\begin{array}{*{20}c} {d_{ii} = \mathop \sum \limits_{n = 1}^{f} \mathop \sum \limits_{j = 1,j \ne i}^{f} W_{ij} } \\ \end{array}$$

Where $D$ represents the diagonal matrix, the diagonal elements are the sum of the weights of the corresponding nodes, $L$ represents the Laplace matrix, and $d_{ii}$ represents the degree of the $i$-th node, that is, the sum of the weights of all its neighboring nodes. Then calculate the symmetric normalized Laplace matrix $L_{sym}$, and the calculation formula is shown in Eq. 13:

13 $$\begin{array}{*{20}c} {L_{sym} = I - D^{{ - \frac{1}{2}}} WD^{{ - \frac{1}{2}}} } \\ \end{array}$$

Where $L_{sym}$ represents the symmetric normalized Laplace matrix, $I$ is the identity matrix, and $D^{{ - \frac{1}{2}}}$ is the inverse square root of the degree matrix, which is used to normalize node weights to prevent nodes with large degrees from excessively influencing their neighbors.

Subsequently, we use orthogonal decomposition to calculate the normalized Laplace matrix, and the calculated eigenvalues are used as node features. The calculation formula for orthogonal decomposition is shown in Eq. 14:

14 $$\begin{array}{*{20}c} {L_{sym} = U\Lambda U^{T} } \\ \end{array}$$

where $\Lambda = diag\left( {\left[ {\lambda_{0} ,\lambda_{1} ,...,\lambda_{d - 1} } \right]} \right)$ represents the eigenvalue diagonal matrix of the Laplacian matrix after orthogonal decomposition, and $U = [u_{0} ,u_{1} ,...,u_{d - 1} ]$ represents the eigenvector matrix of the Laplacian matrix after orthogonal decomposition. Each node feature in the spatio-temporal graph can be represented by a one-dimensional feature vector $x$, which consists of eigenvalues and is expressed as $x = \left[ {\lambda_{0} ,\lambda_{1} , \ldots ,\lambda_{d - 1} } \right]$.

The final generated spatio-temporal graph is $G = (X,A)$, where each node in $V$ represents a bearing fault sample, and $X = \{ x_{1} ,x_{2} ,...,x_{n} \} \in {\mathrm{R}}^{n \times d}$ represents the feature matrix of the bearing fault sample.

### Dual-branch decision network

Single-branch Chebyshev graph convolutional networks have obvious adaptability issues. First, the characteristic distributions of normal and abnormal bearing signals are extremely different, which causes the gradient of fault samples to be dominated by normal samples, resulting in insufficient fault feature learning. Secondly, the characteristics of minor fault samples are masked by major fault samples, causing the model to misclassify minor fault samples as major fault samples. To address these issues, we designed a parameter-independent dual-branch Chebyshev graph convolutional network. By differentially training all features and fault features, we continue to learn the characteristic patterns of fault samples based on the features of all samples, thereby improving the specificity and accuracy of feature extraction.

To ensure that the two branches focus on learning all features and fault features respectively and avoid gradient interference between the two models, we designed a differentiated training mechanism to ensure stable network training. For the normal model, we use all samples in the training set for training, perform gradient updates on the parameters of the normal model through the loss function, and learn the characteristic patterns of all signals. The loss function $L_{nor}$ of the normal model is defined as shown in Eq. 18:

15 $$\begin{array}{*{20}c} {L_{nor} = E_{{i \in V_{train}^{nor} }} \left[ {H\left( {y_{{v_{i} ,c}} ,\hat{y}_{{v_{i} ,c}} } \right)} \right]} \\ \end{array}$$

where $V_{train}^{nor}$ is all the training data used by the normal model, $H( \cdot , \cdot )$ is the cross entropy loss function, $y_{{v_{i} ,c}}$ represents the true label of node $v_{i}$ belonging to category $c$, and $\hat{y}_{{v_{i} ,c}}$ represents the probability predicted by the model that node $v_{i}$ belongs to category $c$.

For the fault model, we use the fault samples in the training set for training, perform gradient updates on the parameters of the fault model through the loss function, and learn the characteristic patterns of fault signals of different categories. The loss function $L_{fal}$ of the fault model is defined as shown in Eq. 16:

16 $$\begin{array}{*{20}c} {L_{fal} = E_{{i \in V_{train}^{fal} }} \left[ {H\left( {y_{{v_{i} ,c}} ,\hat{y}_{{v_{i} ,c}} } \right)} \right]} \\ \end{array}$$

Where $V_{train}^{fal}$ is the fault training data used by the fault model, $H( \cdot , \cdot )$ is the cross-entropy loss function, $y_{{v_{i} ,c}}$ represents the true label of node $v_{i}$ belonging to category $c$, and $\hat{y}_{{v_{i} ,c}}$ represents the probability predicted by the model that node $v_{i}$ belongs to category $c$.

### Decision fusion

The dual-branch Chebyshev graph convolutional network has different emphasis on the data in the training set, resulting in significant differences in the training process of the dual-branch model. Specifically, when the proportion of normal samples in the data set is higher than that of fault samples, the feature learning of fault samples by the normal model is easily dominated by normal samples, resulting in high recognition accuracy for normal samples, but insufficient feature capture ability for minor fault samples. Since the fault model focuses on training fault samples, the recognition accuracy of fault samples is significantly improved, but the features of normal samples are not sufficiently fitted, and normal samples are easily misjudged as minor faults. However, traditional fixed-weight fusion cannot take into account both the recognition accuracy of normal samples and the sensitivity of fault samples, resulting in the accuracy after fusion being lower than the optimal value of a single branch.

To address the aforementioned problems, we propose an adaptive attention-based decision fusion mechanism. Its core adjustment rule is an adaptive allocation mechanism based on sample feature differences, guided by multi-objective loss, and constrained by weight normalization. This mechanism dynamically learns attention weights through a multi-layer perceptron (MLP), differentially weighting the model’s output. This ensures that normal samples are prioritized by the normal model, faulty samples by the fault model, and slightly faulty samples balance the outputs of both models, thus solving the performance bias problem of a single model.

In order to enable MLP to simultaneously utilize the feature information output by the dual-branch network, the output features of the normal model and the fault model need to be spliced in the feature dimension to form a fusion input matrix. The feature splicing formula is shown in Eq. 17:

17 $$\begin{array}{*{20}c} {C = \left[ {O_{nor} ,O_{fal} } \right]} \\ \end{array}$$

Among them, $O_{nor} \in {\mathrm{R}}^{N \times C}$ is the output feature matrix of the normal model, $O_{fal} \in {\mathrm{R}}^{N \times C}$ is the output feature matrix of the fault model, and $C \in {\mathrm{R}}^{N \times 2C}$ is the concatenated fusion input matrix. Each row of the fusion input matrix $C$ corresponds to the normal branch feature and fault branch feature of a node, and contains the complete feature basis for determining the label category to which the node belongs.

In order to dynamically allocate attention weights, we use MLP to map the $2C$-dimensional fusion input matrix into a 2-dimensional attention weight vector, and normalize the attention weights through the softmax function to obtain the final attention weight matrix. The calculation of the attention weight matrix $\omega_{att}$ is shown in Eq. 18:

18 $$\begin{array}{*{20}c} {X^{l} = f\left( {W^{l - 1} X^{l - 1} + b^{l - 1} } \right)} \\ \end{array}$$

Where $X^{0} = C$ represents the fused input matrix of layer 0, $f\left( \cdot \right)$ represents the activation function, such as ReLU, Sigmoid, etc., $W^{l - 1}$ is the weight matrix of layer $l - 1$, $b^{l - 1}$ is the bias of layer $l - 1$, and $X^{l} \in {\mathrm{R}}^{N \times 2}$ is the unnormalized attention weight. Then, $X^{l}$ is normalized to the interval [0,1] through the Softmax function to obtain the attention weight matrix $\omega_{att}$.

Based on the attention weight matrix $\omega_{att}$, the output features of the two-branch Chebyshev network are weighted and summed to obtain the final fusion feature matrix $O_{fus}$. The calculation formula for weighted feature fusion is shown in Eq. 19:

19 $$\begin{array}{*{20}c} {O_{fus} = \omega_{att}^{nor} \cdot O_{nor} + \omega_{att}^{fal} \cdot O_{fal} } \\ \end{array}$$

Where $O_{fus} \in {\mathrm{R}}^{N \times C}$ is the final fusion feature matrix, $\omega_{att}^{nor}$ and $\omega_{att}^{fal}$ represent the weights of the normal model and fault model output, respectively. The obtained fusion feature vector $O_{fus}$ is converted into a category probability distribution through the log_softmax activation function, and the fusion loss function is calculated. The fusion loss function $L_{fus}$ is defined as shown in Eq. 20:

20 $$\begin{array}{*{20}c} {L_{fus} = E_{{i \in V_{train} }} \left[ {H\left( {y_{{v_{i} ,c}} ,\hat{y}_{{v_{i} ,c}} } \right)} \right]} \\ \end{array}$$

Where $V_{train}$ is the training data used by the MLP model, $H( \cdot , \cdot )$ is the cross-entropy loss function, $y_{{v_{i} ,c}}$ represents the true label of node $v_{i}$ belonging to category $c$, and $\hat{y}_{{v_{i} ,c}}$ represents the probability that node $v_{i}$ belongs to category $c$ after fusion.

In order to simultaneously optimize the feature fitting capabilities of the multi-layer perceptron, normal model, and fault model, and avoid the degradation of model performance caused by single-objective optimization, we designed a multi-objective loss function to optimize the model. The calculation formula of the multi-objective loss function is shown in Eq. 21:

21 $$\begin{array}{*{20}c} {L_{total} = L_{fus} + \alpha \cdot L_{nor} + \left( {1 - \alpha } \right) \cdot L_{fal} } \\ \end{array}$$

Where $\alpha$ is the weight coefficient.

### Computational complexity analysis

We performed a complexity analysis of the DBSGN in this study. The DBSGN mainly consists of three parts: spatio-temporal graph generation, a dual-branch decision network, and decision fusion. Therefore, the computational cost also comes from these three parts. In the spatio-temporal graph generation stage, the complexity of the short-time Fourier transform is $O{ }\left( {LTlogT} \right)$, the calculation of the adjacency weight matrix is $O{ }\left( {N^{2} d} \right)$, and the orthogonal eigenvalue decomposition of the Laplacian matrix is $O{ }\left( {N^{3} } \right)$; the dual-branch decision network contains $K$ convolutional layers, the complexity of a single branch is $O{ }\left( {KNd^{2} } \right)$, and the total complexity of the dual-branch is $O{ }\left( {KNd^{2} } \right)$; the complexity of feature concatenation, weight learning, and fusion calculation in the decision fusion part is low and can be ignored. The overall time complexity is $O{ }\left( {N^{3} + KNd^{2} } \right)$. In sparse graphs or practical scenarios, when $N \gg d$ and $N \gg K$, the dominant term is $O{ }\left( {N^{3} } \right)$. The spatial complexity is mainly dominated by the storage of the $N \times N$ adjacency matrix, which is $O{ }\left( {N^{2} } \right)$, while the storage cost of the feature matrix and network parameters is negligible. The pseudo code of the model is shown in Algorithm 1.

**Algorithm 1:**
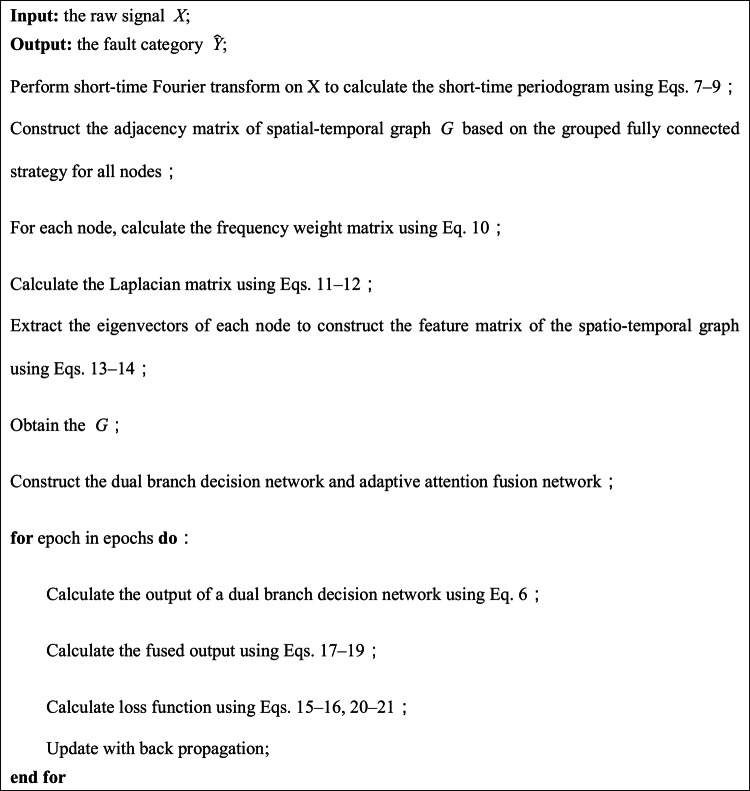
Dual-branch space–time graph network(DBSGN).

## Experiment

### Experimental setup

*Dataset* To evaluate the proposed model, we conduct extensive experiments on data provided by Case Western Reserve University (CWRU)^44^, University of Paderborn (PU)^45^, and University of Ottawa (uOttawa)^46^. The details of the fault dataset are shown below.

*CWRU bearing dataset*^44^: The CRWU dataset is provided by Case Western Reserve University and is a public dataset widely used in the field of rolling bearing fault diagnosis. This dataset is bearing test data obtained from an experimental test bench, which consists of a 2HP induction motor, a torque sensor, a dynamometer, and a deep groove ball bearing. Vibration data is captured using accelerometers installed at different positions on the motor housing.

The experiment collected data under four different motor load conditions: 0HP, 1HP, 2HP, and 3HP, and the vibration signal was sampled at a frequency of 12 kHz. The 12 k drive-end bearing failure data includes four failure diameters: 0.007 inches, 0.014 inches, 0.021 inches, and 0.028 inches. The 0.028 inches uses an NTN-6205-2RS equivalent bearing, and the others all use SKF-6205-2RS bearings. Each fault diameter includes three fault types: inner race (IR), outer race (OR), and ball (B), with the outer race fault data missing for 0.028 inches. Based on the above, we constructed a comprehensive dataset containing 12 different bearing health conditions: 1 normal condition and 11 fault conditions. Table 1 summarizes the details of the dataset composition.

**Table 1:** Composition of the CWRU rolling bearing experimental dataset.

No.	Fault diameter	Fault location	Fault class	Label	Samples size
1	0	Normal	0	0	200
2	0.007	Inner raceway	IR007	1	200
3		Ball	B007	2	200
4		Outer raceway	OR007	3	200
5	0.014	Inner raceway	IR014	4	200
6		Ball	B014	5	200
7		Outer raceway	OR014	6	200
8	0.021	Inner raceway	IR021	7	200
9		Ball	B021	8	200
10		Outer raceway	OR021	9	200
11	0.028	Inner raceway	IR028	10	200
12		Ball	B028	11	200

*PU bearing dataset*^45^: The PU dataset is published by Paderborn University. Its core goal is to provide support for the research and application of data-driven classification methods. It is mainly used to monitor and diagnose bearing damage in electromechanical drive systems through motor current signals. This dataset contains bearing test data obtained from an experimental test bench. The test bench consists of a drive motor, a torque measurement shaft, a rolling bearing experimental module, a flywheel, and a load motor. The test bench obtains signal data under different working conditions by applying different loads and speeds to the experimental bearings.

The sampling frequency of the vibration signal in this dataset is 64 kHz. Each bearing fault data contains four operating conditions. The detailed information of different operating conditions is shown in Table 2. The dataset for each working condition contains three fault types: healthy, inner loop fault, and outer loop fault. Based on the above, we constructed a dataset containing four working conditions, each of which includes three fault types.

**Table 2:** Working condition information of PU rolling bearing experimental dataset.

No.	Rotational speed [rpm]	Load torque [Nm]	Radial force [N]	Name of setting	Samples size
1	1500	0.7	1000	N15_M07_F10	300
2	900	0.7	1000	N09_M07_F10	300
3	1500	0.1	1000	N15_M01_F10	300
4	1500	0.7	400	N15_M07_F04	300

*uOttawa bearing dataset*^46^: The uOttawa dataset is provided by the University of Ottawa. The experiments were conducted on a SpectraQuest Mechanical Fault Simulator (MFS-PK5M), which consists of an AC drive, a motor, two ER16K ball bearing support shafts, and bearings. Vibration data was collected by placing an ICP accelerometer on the housing of the experimental bearing. In addition, an EPC incremental encoder was installed to measure shaft speed.

The data contains vibration signals collected from bearings in different health conditions under time-varying speed conditions. The vibration signals are sampled at a frequency of 200 kHz. Each dataset contains two experimental settings: bearing health condition and speed change condition. The health status of a bearing includes health, inner ring defect failure, outer ring defect failure, ball defect failure, and combined defect failure of the inner ring, outer ring and ball. The operating speed conditions are increasing speed, decreasing speed, increasing speed and then decreasing speed, and decreasing speed and then increasing speed. Based on the above, we constructed a comprehensive dataset containing five different bearing health states: one normal condition and four fault conditions. Table 3 summarizes the details of the dataset composition.

**Table 3:** Composition of the uOttawa rolling bearing experimental dataset.

Bearing health conditions	Speed varying conditions				Samples size
	Increasing speed	Decreasing speed	Increasing then decreasing speed	Decreasing then increasing speed	
Healthy	H-A-1	H-B-1	H-C-1	H-D-1	600
	H-A-2	H-B-2	H-C-2	H-D-2	
	H-A-3	H-B-3	H-C-3	H-D-3	
Inner race defect	I-A-1	I-B-1	I-C-1	I-D-1	600
	I-A-2	I-B-2	I-C-2	I-D-2	
	I-A-3	I-B-3	I-C-3	I-D-3	
Outer race defect	O-A-1	O-B-1	O-C-1	O-D-1	600
	O-A-2	O-B-2	O-C-2	O-D-2	
	O-A-3	O-B-3	O-C-3	O-D-3	
Ball defect	B-A-1	H-B-1	B-C-1	B-D-1	600
	B-A-2	B-B-2	B-C-2	B-D-2	
	B-A-3	B-B-3	B-C-3	B-D-3	
Combined defects on the inner race, the outer race and a ball	C-A-1	C-B-1	C-C-1	C-D-1	600
	C-A-2	C-B-2	C-C-2	C-D-2	
	C-A-3	C-B-3	C-C-3	C-D-3	

*Baseline* To demonstrate the advantages of our model, this study selected some representative and popular methods in the field of fault diagnosis as baseline models for comparison with our proposed method, as shown below:

*Convolutional Neural Network (CNN)*^47^: CNN is a benchmark method in deep learning. In this experiment, bearing signals are predicted by converting them into image information.

*SuperGraph*^32^: SuperGraph converts raw data into a hypergraph through short-time Fourier transform (STFT) and uses graph neural networks to predict nodes in the hypergraph.

*WPT-FFT*^48^: This method decomposes the vibration signal through wavelet packet transform (WPT) and Fourier transform (FFT), extracts high-resolution features from the short-term vibration signal, and uses the Bayesian optimized random forest algorithm for classification.

*Multi-scale CNN-Transformer*^49^: This method uses the whale optimization algorithm to determine the optimal parameters for continuous variational mode decomposition. After reconstructing the signal using intrinsic mode functions, a multi-scale convolutional neural network and a Transformer are used to extract local and global features, thereby achieving fault type classification.

*MultiPatchTST*^50^: This method utilizes multiple PatchTST models based on the Transformer architecture to generate different intermediate results, and then uses an MLP layer to generate the final prediction.

*HMDN*^51^: HMDN employs a tree-structured labeling scheme to annotate multi-granularity faults and improves intra-class compactness and reduces inter-class hierarchical similarity through a hierarchical multi-granularity diagnostic network and a multi-granularity fault loss function.

*Evaluation indicators* To evaluate the effectiveness of our model, this experiment selected accuracy, precision, recall, F1-score, AUC curve, and Matthews correlation coefficient (MCC) as evaluation indicators to measure the performance of the model in the bearing fault multi-classification task. Among them, accuracy is the basic indicator that reflects the overall correct recognition ratio of the model for all samples. Precision, recall, and F1 score judge the performance of the model from the perspective of category balance and overall sample recognition effect. The AUC curve and MCC can more robustly evaluate the classification stability of the model in data imbalance scenarios. By considering these six evaluation indicators, the evaluation bias caused by category shift of a single indicator can be avoided.

*Detailed experimental setup* For all experiments in this study, we refer to some previous experimental setups^32,42,48^. Specifically, for the CWRU, PU, and uOttawa datasets, we did not add any noise, meaning the signal-to-noise ratio level is 0 dB. Then, we used stratified sampling to divide the data into training, validation, and test sets, with proportions of 60%, 20%, and 20%, respectively. This method ensures that the category distribution of each dataset is consistent with the original data, thus avoiding class imbalance, and also guarantees that the test samples are completely independent of the training samples, preventing the risk of temporal data leakage. Subsequent spatio-temporal graph construction and model training were performed based on the training set for parameter learning. After model training, the trained model was tested using the test set, ensuring no cross-set information leakage throughout the entire process. In addition, all experiments were repeated 10 times independently, and the model parameters were reinitialized in each experiment. The final result was the mean of the 10 experiments to avoid the influence of random errors in a single experiment on the conclusion. The training and evaluation of all experiments were performed on a computer with an Intel(R) Core(TM) i7-10750H CPU @ 2.60 GHz, an NVDIA GTX 1650 GPU, a Kingston 32 GB memory, and Windows 10 Professional. The model in this study is based on Python 3.9 (1) and is developed using open-source libraries such as PyTorch 2.2 (2), scikit-learn 1.5.2 (3), and NumPy 1.26.3 (4).

1. https://www.python.org/downloads/release/python-390/
2. https://pytorch.org/blog/pytorch2-2/
3. https://scikit-learn.org/1.5/whats_new/v1.5.html
4. https://numpy.org/doc/2.1/release/1.26.3-notes.html

### Comparison with other advanced methods

To evaluate the performance of our proposed DBSGN on three bearing fault datasets, we selected six bearing fault diagnosis methods for comparative experiments with our method. We also calculated the performance of different bearing fault diagnosis methods under six evaluation indicators. The experimental results are shown in Table 4.

**Table 4:** Performance of different models under six evaluation metrics in three datasets.

Dataset	Model	Evaluation metric					
		Accuracy	Precision	Recall	F1-score	AUC	MCC
CWRU	CNN	99.72	99.72	99.72	99.72	99.99	99.58
	SuperGraph	98.54	98.43	98.51	98.44	99.90	98.42
	WPT-FFT	99.66	99.59	99.59	99.59	100.00	99.62
	multi-scale CNN-Transformer	99.24	99.16	99.16	99.16	99.98	99.25
	MultiPatchTST	99.42	99.73	99.54	99.63	99.99	99.69
	HMDN	96.67	96.94	96.67	96.72	97.99	96.32
	DBSGN	**99.79**	**99.80**	**99.85**	**99.82**	**100.00**	**99.77**
PU	CNN	76.10	77.31	76.05	75.86	94.78	74.07
	SuperGraph	97.22	97.44	96.67	96.91	99.88	95.90
	WPT-FFT	95.83	95.87	95.83	95.80	99.53	93.80
	multi-scale CNN-Transformer	99.68	99.73	99.74	99.73	99.99	99.88
	MultiPatchTST	98.32	98.63	98.89	98.75	99.94	98.74
	HMDN	96.22	96.25	96.22	96.22	97.83	96.10
	DBSGN	**100.00**	**100.00**	**100.00**	**100.00**	**100.00**	**100.00**
uOttawa	CNN	91.06	92.17	91.06	90.80	99.04	89.20
	SuperGraph	97.50	97.59	97.58	97.49	99.68	96.93
	WPT-FFT	99.17	99.17	99.17	99.16	99.99	98.96
	multi-scale CNN-Transformer	99.58	99.59	99.58	99.58	99.99	99.57
	MultiPatchTST	98.67	98.67	98.67	98.67	99.78	98.52
	HMDN	96.22	96.25	96.22	96.22	98.78	96.10
	DBSGN	**99.83**	**99.83**	**99.84**	**99.84**	**100.00**	**99.79**

Bold and underlined lines indicate the best and second best results, respectively.

Experimental results show that compared to bearing fault diagnosis methods such as CNN, SuperGraph, WPT-FFT, multi-scale CNN-Transformer, and MultiPatchTST, the proposed DBSGN model significantly improves fault diagnosis performance on these three datasets. On the CWRU dataset, compared to the baseline models, the accuracy of the DBSGN model increased by approximately 0.07%, and the precision, recall, F1-score, and MCC also increased by 0.07%, 0.13%, 0.1%, and 0.15%, respectively. On the PU dataset, all evaluation metrics of the DBSGN model reached 100.00%, showing a significant improvement compared to other baseline models. This indicates that our model has efficient recognition capabilities for this bearing fault dataset, even achieving zero-error recognition of fault samples on this dataset. Furthermore, on the uOttawa dataset, compared to the best baseline model multi-scale CNN-Transformer, the accuracy of the DBSGN model increased by approximately 0.66%, and the precision, recall, F1-score, AUC, and MCC also increased by 0.66%, 0.67%, 0.67%, 0.01%, and 0.83%, respectively.

### Further analysis

**Fig. 2:**
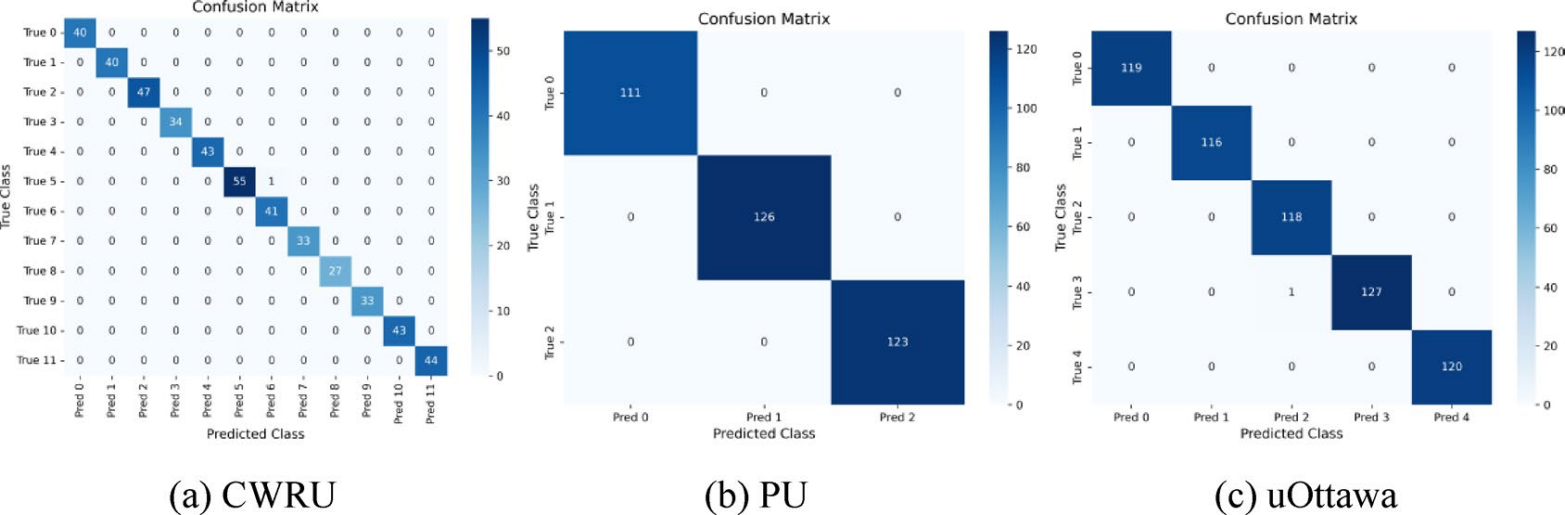
Confusion matrix of three datasets.

**Fig. 3:**
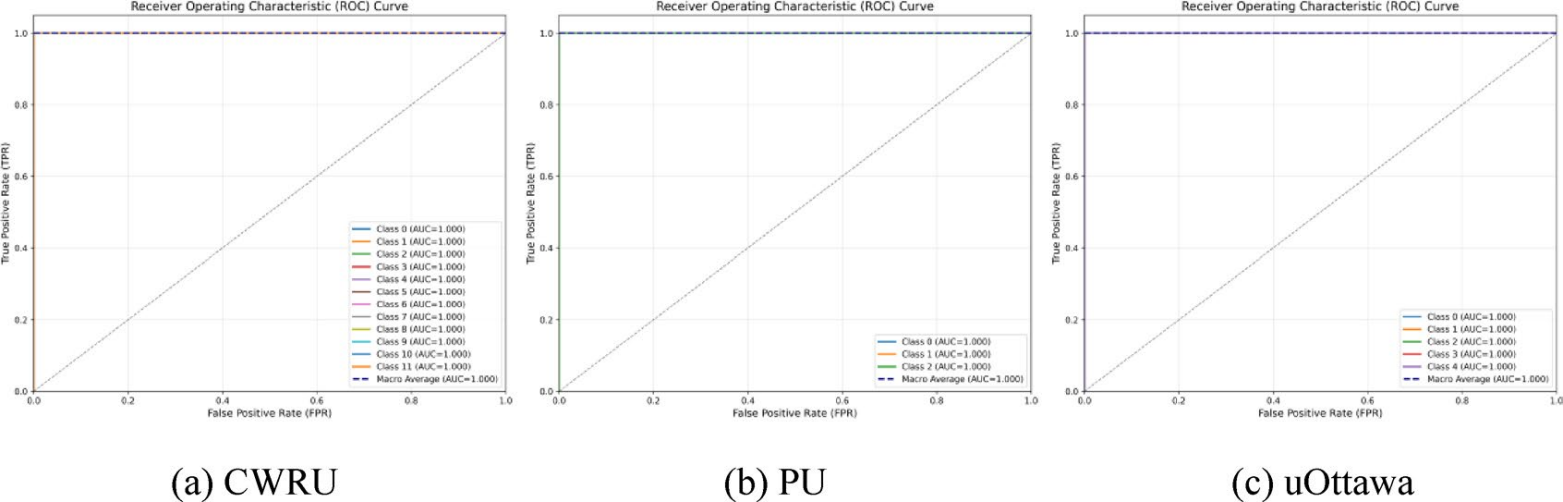
ROC curves of three datasets.

**Fig. 4:**
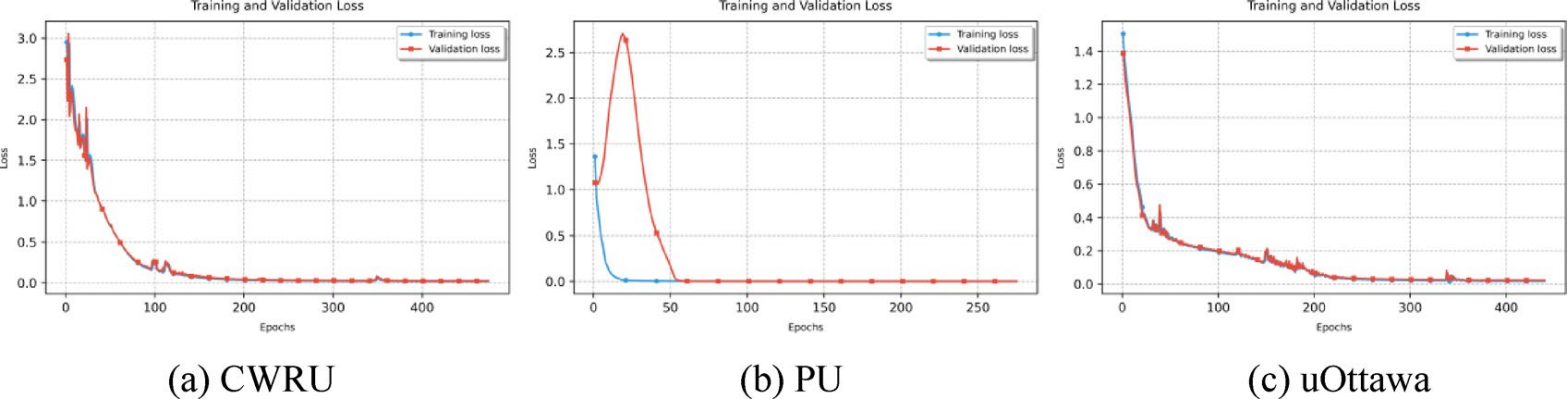
Loss function convergence curves of three datasets.

To further validate the performance of our proposed DBSGN model in bearing fault detection, this paper conducts a detailed analysis of the model’s class discrimination ability and probability prediction reliability from three perspectives: confusion matrix, ROC curve, and convergence curve. The experimental results are shown in Figs. 2, 3 and 4.

In the confusion matrix of Fig. 2, each row corresponds to the true category label, each column corresponds to the predicted category label, and the diagonal elements represent the number of correctly classified samples for each type of bearing condition. In the confusion matrix of the CWRU dataset, the number of samples on the main diagonal accounts for a very high proportion, while the number of samples on the off-diagonal accounts for a very low proportion, indicating that the DBSGN model has high recognition accuracy for various types of faults in the CWRU dataset, and the vast majority of samples can be correctly classified into the corresponding categories. In the confusion matrix of the PU bearing dataset, all samples are completely concentrated on the main diagonal, indicating that the model can achieve zero-error classification performance for the faulty samples in this dataset. In the confusion matrix of the uOttawa dataset, only a very small number of samples have cross-category prediction bias, verifying that the model still maintains high classification accuracy in multi-category, fine-grained fault scenarios.

From the ROC curve results in Fig. 3, it can be seen that the ROC curves of the three bearing data sets are all close to the upper left corner of the coordinate system, and the AUC values ​​of each category and macro average are all 1. This shows that the model’s ability to distinguish between positive and negative categories for each type of fault has reached the theoretical optimal level, and the discriminatory degree of probability prediction is extremely strong. It can achieve perfect distinction between faults and non-faults, as well as between different faults, in data sets with different numbers of categories and different fault degrees.

Figure 4 shows the training and validation loss convergence curves of the proposed model on the CWRU, PU, and uOttawa bearing datasets. In the CWRU dataset’s loss convergence curve, both training and validation losses decrease rapidly with the number of iterations and then stabilize, with minimal fluctuations and slight numerical differences. This indicates that the model’s fitting process is stable on this dataset, without significant overfitting or underfitting issues. In the uOttawa dataset’s loss convergence curve, both training and validation losses decrease rapidly initially and then remain at a low level. Although the validation loss shows slight fluctuations, the difference between it and the training loss remains small and the trends are consistent, verifying the model’s stable convergence performance in multi-class, fine-grained fault scenarios. In the PU bearing dataset’s loss convergence curve, the training loss converges rapidly to a stable level in the initial iterations, and the validation loss fluctuates briefly before decreasing to near zero and remaining stable. The two show highly consistent trends in the later stages of training, reflecting the model’s fast convergence speed and strong generalization ability.

Based on the analysis of confusion matrix and ROC curve, the DBSGN model proposed in this paper demonstrates high-precision and high-robustness classification performance in bearing fault diagnosis tasks, and can provide reliable technical support for bearing health monitoring in industrial scenarios.

### Ablation study

In this section, we conduct detailed ablation experiments to evaluate the effectiveness of the proposed two-branch decision network. The rest of the experimental settings are consistent with those used in our previous comparative experiments.

**Fig. 5:**
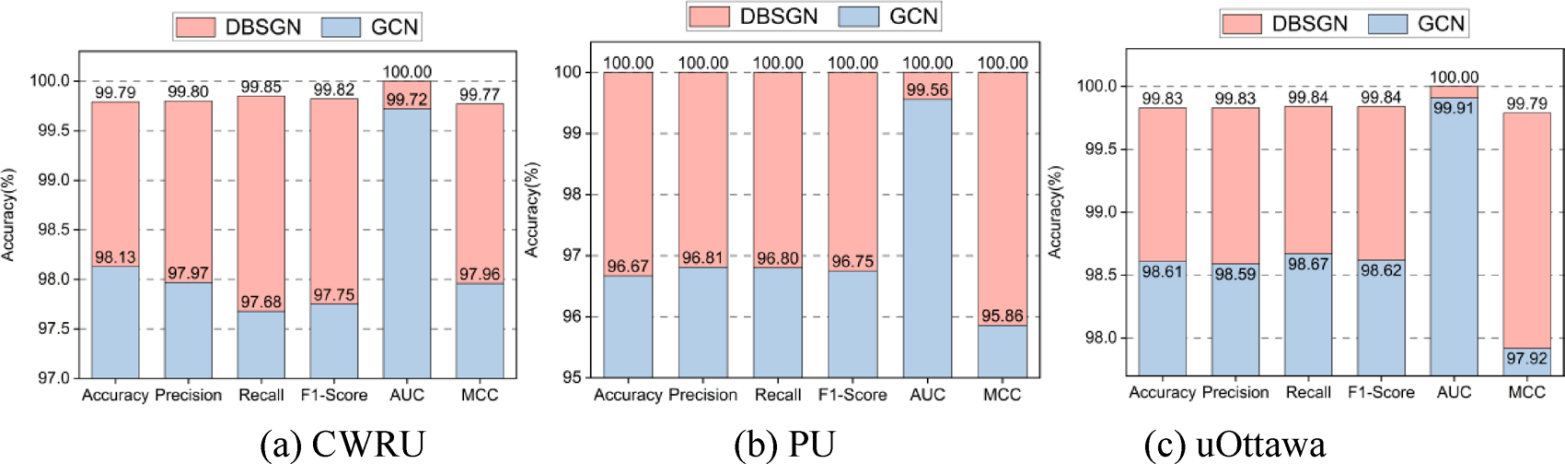
Ablation study.

In Sects “Dual-branch decision network” and “Decision fusion”, we proposed a dual-branch decision network, fused the dual-branch output features through an adaptive attention decision fusion mechanism, and performed fault prediction based on the fusion results. To verify the effectiveness of the algorithm, a single Chibyshev graph convolutional network is used as a comparison method. The network removes the dual-branch structure and adaptive fusion mechanism and directly performs single-branch graph convolution prediction on graph data. Then, the fault prediction performance of the dual-branch decision network and the single network for vibration signals is evaluated on three bearing fault datasets: CWRU, PU and uOttawa. Figure 5 demonstrates the importance of the dual-branch decision network. Experimental results show that on the CWRU dataset, our proposed DBSGN improves accuracy, precision, and recall by approximately 2% compared to GCN. On the PU dataset, DBSGN’s accuracy, precision and other indicators all reached 100%, significantly outperforming the performance of GCN. On the uOttawa dataset, DBSGN achieves significant improvements in core indicators compared to GCN, with an average improvement of about 1.2%. This fully proves that the dual-branch decision network combined with the adaptive fusion mechanism is effective and can significantly enhance the recognition ability of bearing fault characteristics through differentiated feature learning and dynamic weight allocation.

We further analyzed the differences in computational complexity and efficiency of the model before and after ablation, and the experimental results are shown in Table 5. Table 5 compares the FLOPs, number of parameters, training time per sample, and time per iteration before and after ablation on the CWRU, PU, and uOttawa bearing fault datasets. The results show that the number of parameters, FLOPs, and training time per sample of DBSGN are all increased compared to GCN, and the increase varies with the heterogeneity of the dataset. The increase is most significant in the PU dataset, and the iteration time only increases in the PU dataset, remaining stable in CWRU and uOttawa. The increased computational cost brings significant gains in model performance. The core metrics on the CWRU dataset improved by approximately 2%, the accuracy on the PU dataset reached 100%, and the core metrics on the uOttawa dataset improved by an average of approximately 1.2%. This confirms the reasonable trade-off between performance and cost of the dual-branch structure and adaptive fusion mechanism. Further optimization of the lightweight design of the branches and batch processing strategies can be explored to balance model performance and computational efficiency, considering the graph structure characteristics of the datasets.

**Table 5:** Computational complexity and efficiency of the model before and after ablation.

Datasets	Model	FLOPs/M	Sample time/s	Iteration time/s	Total parameters
CWRU	GCN	41.330	0.00002	0.020	6187
	DBSGN	133.710	0.00657	0.020	21,642
PU	GCN	1.900	0.00003	0.007	5503
	DBSGN	382.260	0.01122	0.034	44,792
uOttawa	GCN	48.400	0.00332	0.020	5655
	DBSGN	157.510	0.00642	0.020	20,060

### Parameter sensitivity analysis

**Fig. 6:**
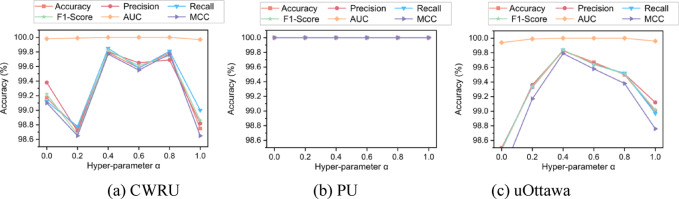
Parameter sensitivity analysis.

In order to study the effect of the hyperparameter $\alpha$ in Eq. 21 on the performance of DBSGN for bearing fault diagnosis, we set the variation range of $\alpha$ to 0–1 with an increment of 0.2, and the experimental results are shown in Fig. 6 Note that the other experimental settings remain the same as those used in the previous experiments.

Experimental results show that on the CWRU and uOttawa datasets, when $\alpha$ is 0.4, the model’s accuracy, precision, recall and other indicators reach the best. After deviating from this value, the indicators show a downward trend. However, on the PU dataset, when $\alpha$ varies from 0 to 1, the results of each evaluation indicator are all 100%. This phenomenon shows that in the CWRU and uOttawa datasets, the performance of the DBSGN model is more sensitive to $\alpha$ and needs to be fine-tuned to match the data distribution characteristics. In the PU dataset, however, changes in the hyperparameter $\alpha$ have no effect on the model. This may be because the data characteristics such as fewer fault samples and strong fault mode discrimination make the model less sensitive to $\alpha$. Based on the experimental results of the above parameter sensitivity analysis, this paper finally sets the hyperparameter $\alpha$ in Eq. 21 to 0.4.

### Visualize feature distribution

**Fig. 7:**
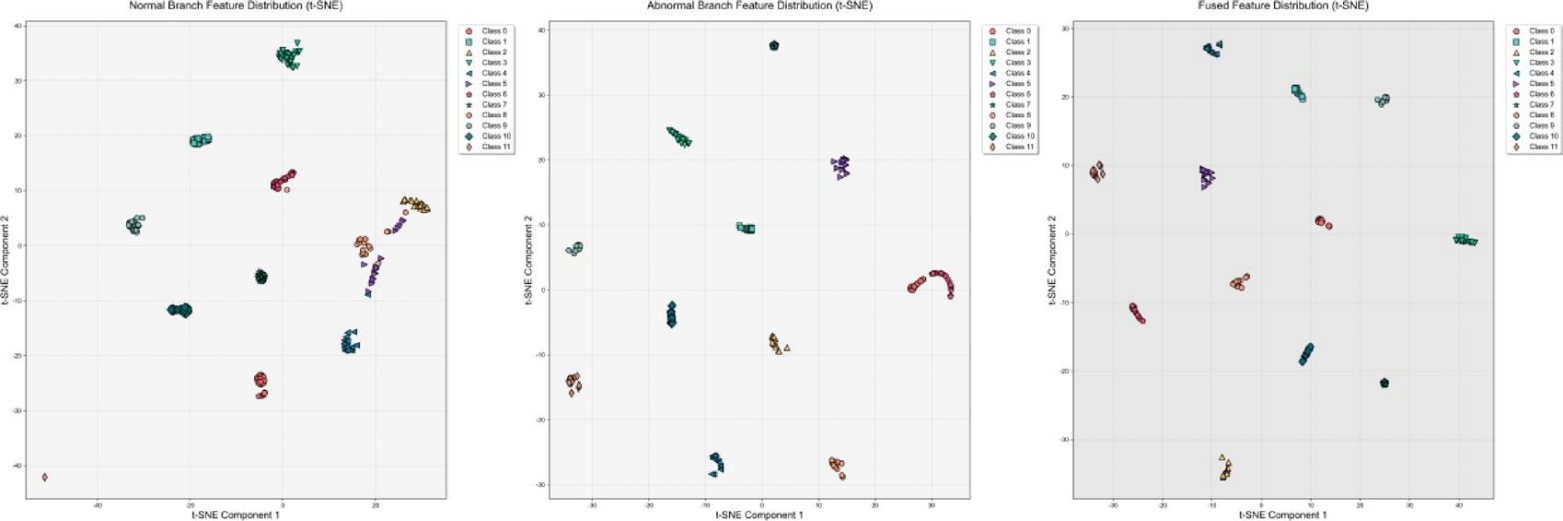
T-SNE feature visualization of the CWRU dataset.

**Fig. 8:**
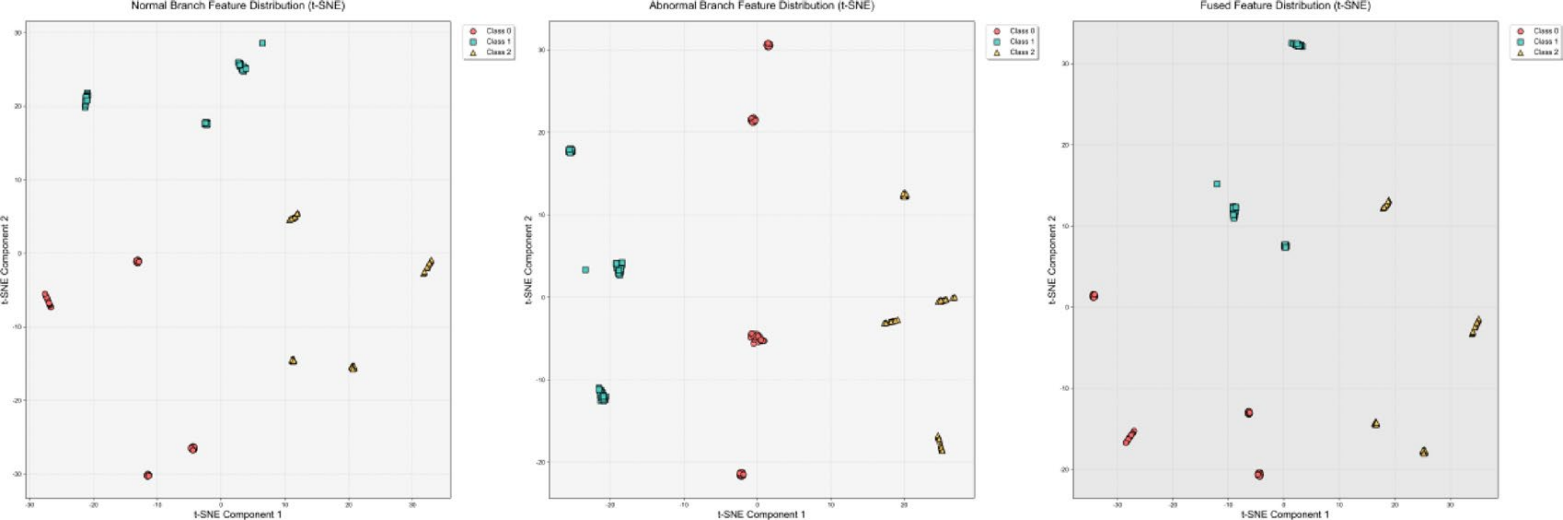
T-SNE feature visualization of the PU dataset.

**Fig. 9:**
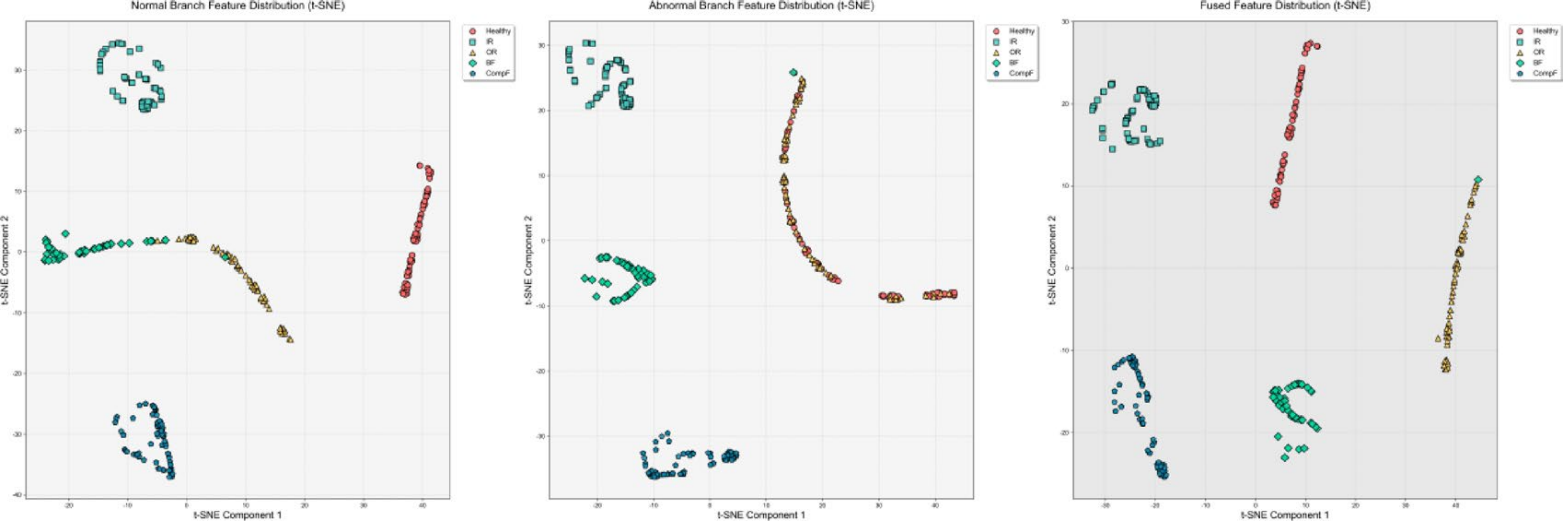
T-SNE feature visualization of the uOttawa dataset.

To intuitively verify the capabilities of the dual-branch decision network and the dynamic attention-based decision fusion mechanism in feature learning, we used the t-SNE visualization method to analyze the feature distributions of the CWRU, PU, and uOttawa datasets. The experimental results are shown in Figs. 7, 8 and 9.

Experimental results show that in the CWRU dataset, the normal branch can only achieve preliminary class separation for 12 types of samples, with significant overlap between feature clusters corresponding to similar faults; although the abnormal branch can strengthen the clustering of fault samples, it causes the normal samples to be scattered. By using an adaptive attention-based decision fusion mechanism to fuse the output features of the dual branches, the feature clusters of each category are completely separated, and the inter-class boundaries are clearly distinguishable. For the PU dataset, the results from the normal branch show marginal overlap in the feature clusters of a few fault categories, while the abnormal branch effectively improves the feature separability of all categories. After fusion, the feature clusters of each category are perfectly separated, and this feature distribution characteristic is highly consistent with the 100% classification accuracy achieved by the model. In the uOttawa dataset, the results from the normal branch show feature cluster crossover among fault categories. Although the abnormal branch eliminates this crossover problem, it leads to a wider distribution range of the healthy sample cluster. After fusion, the feature clusters of each category achieve completely independent distribution, and the feature separability reaches the optimal level. In summary, the normal branch excels at learning and representing global features, while the abnormal branch focuses on the precise extraction of fault features. The decision fusion mechanism proposed in this study effectively integrates the learning advantages of both branches for normal and fault features, resulting in fused features that possess optimal intra-class clustering and inter-class separability. This fully validates the rationality and effectiveness of the DBSGN model structure design.

## Conclusion and future work

This study proposes a dual-branch graph fusion network based on spatio-temporal graphs, which effectively solves the problems of insufficient stability and accuracy in the case of small sample data. The model first models the vibration signal based on spectral graph theory and spectral analysis method to construct a spatio-temporal graph. Secondly, Laplace-based spectral decomposition is used to extract the feature vectors of samples in the spatio-temporal graph. Based on the constructed spatio-temporal graph, we designed a dual-branch fusion network to train and verify the bearing data, and adjusted the model’s learning of the bearing data through a dynamic attention mechanism. Extensive experiments show that the proposed model outperforms traditional models in terms of stability and accuracy compared with other models, verifying the effectiveness of the proposed method in fault diagnosis.

In this paper, our research mainly focuses on how to construct the vibration signal of the bearing into a graph data structure, but the construction of the graph data structure depends on manually preset thresholds. In addition, Laplace spectral decomposition has limited ability to characterize vibration signals. Therefore, in future work, new frequency domain feature extraction methods and graph data structure construction methods can be proposed to improve the model’s representation of vibration signals.

## Supplementary Information

Below is the link to the electronic supplementary material.

Supplementary Material 1

## Data Availability

The datasets generated or analysed during the current study are not publicly available due [Protect the intellectual property rights and commercial interests of investors] but are available from the corresponding author on reasonable request.
