# Supplementary figures and images for: Dual-branch spatio-temporal graph network for bearing fault diagnosis
Source: Sci Rep. 2026 Mar 11;16:13184. doi: 10.1038/s41598-026-42504-0 (PMC13102955; doi:10.1038/s41598-026-42504-0)

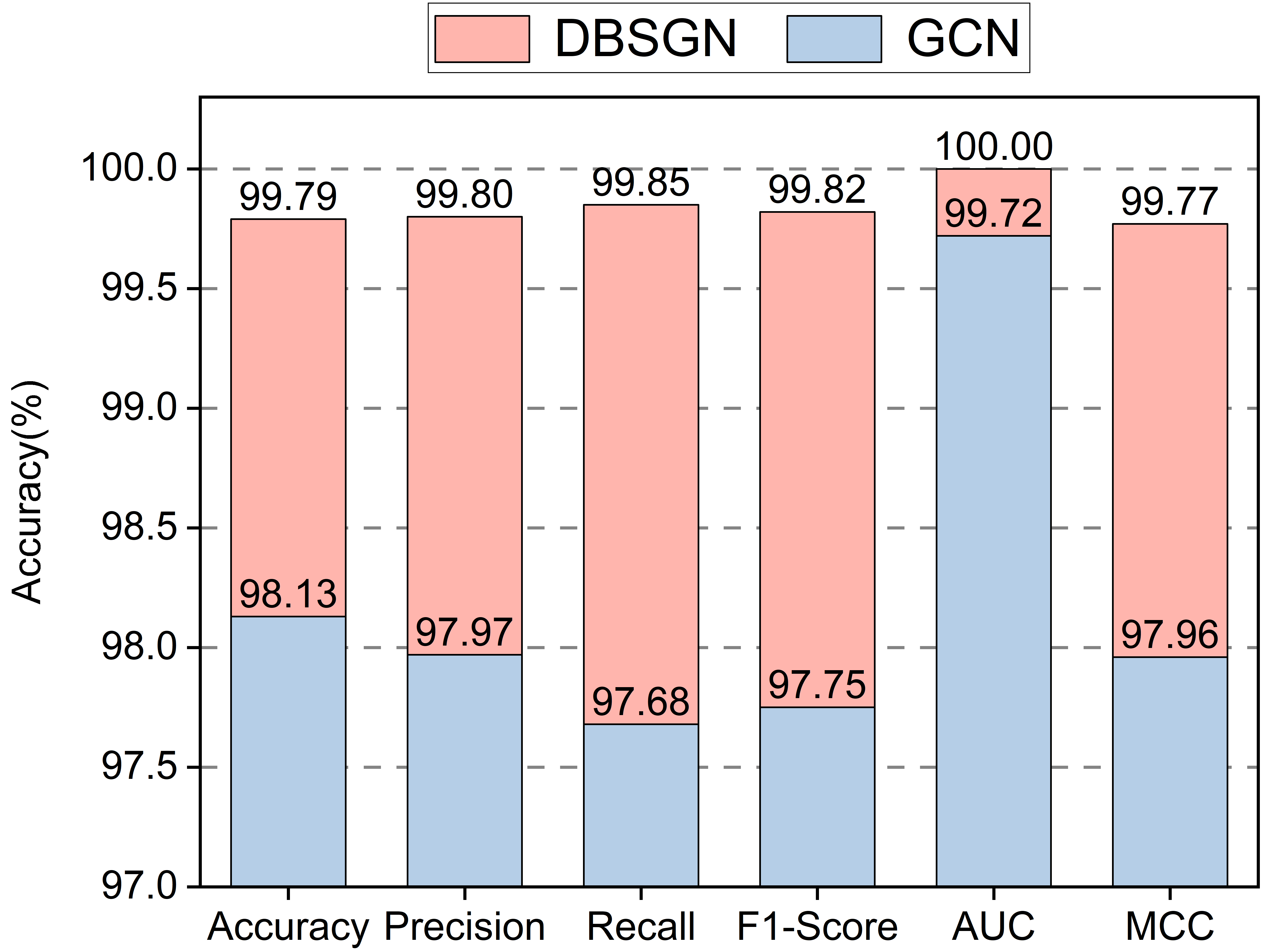

Supplement: Supplementary file 1 — Supplementary Material 1 [file 41598_2026_42504_MOESM1_ESM.zip › Supplementary/Ablation_CWRU.png]

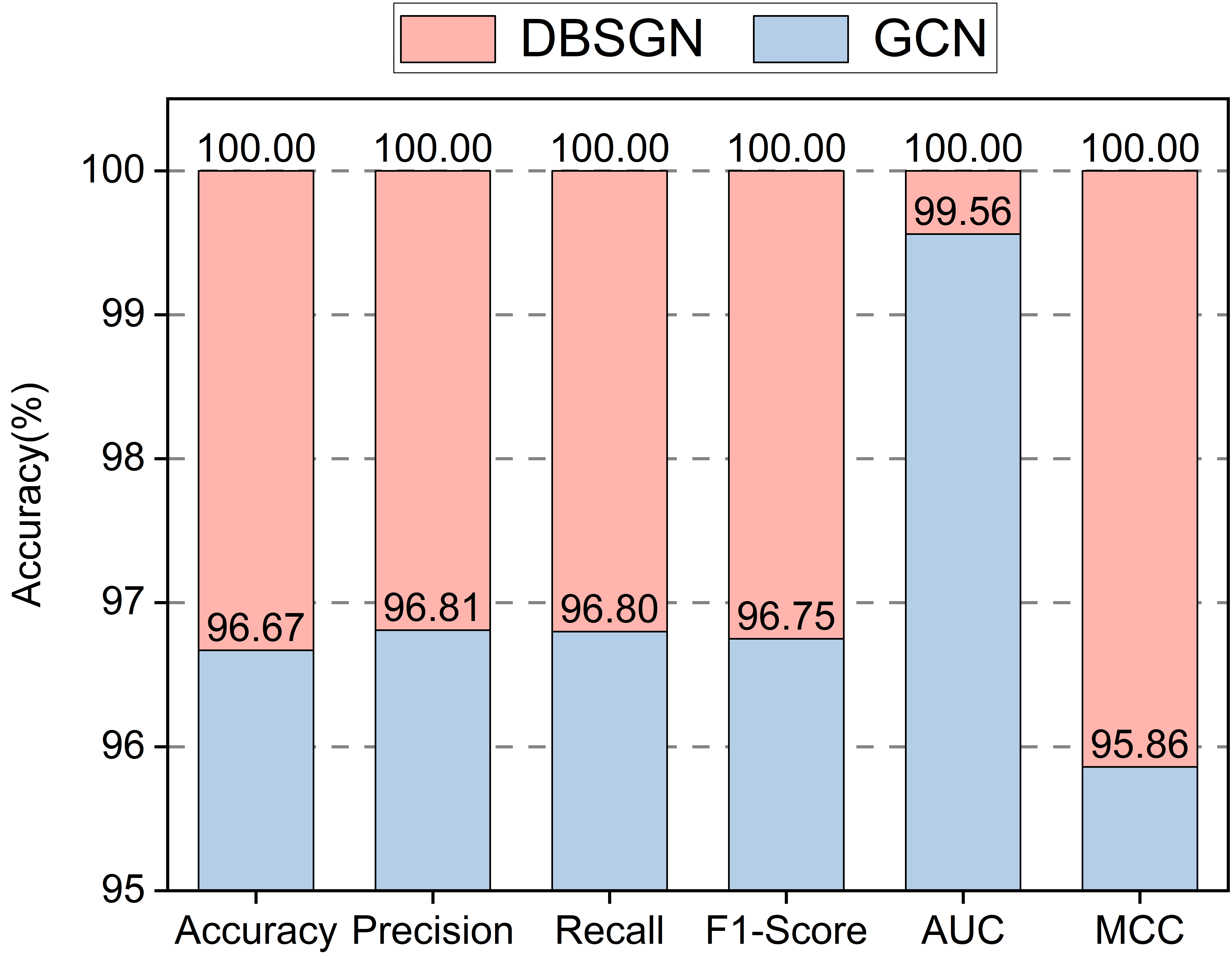

Supplement: Supplementary file 1 — Supplementary Material 1 [file 41598_2026_42504_MOESM1_ESM.zip › Supplementary/Ablation_PU.png]

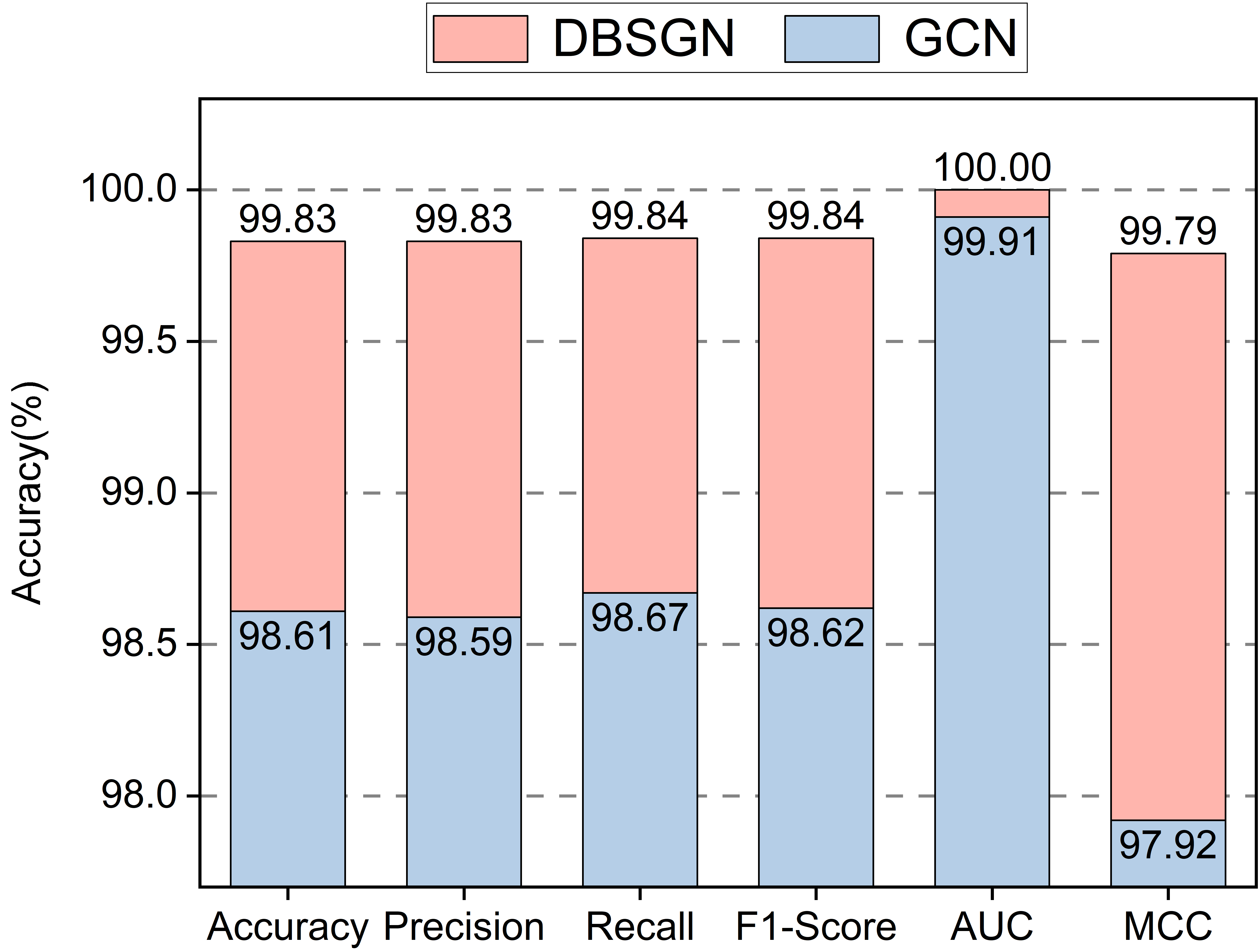

Supplement: Supplementary file 1 — Supplementary Material 1 [file 41598_2026_42504_MOESM1_ESM.zip › Supplementary/Ablation_uOttawa.png]

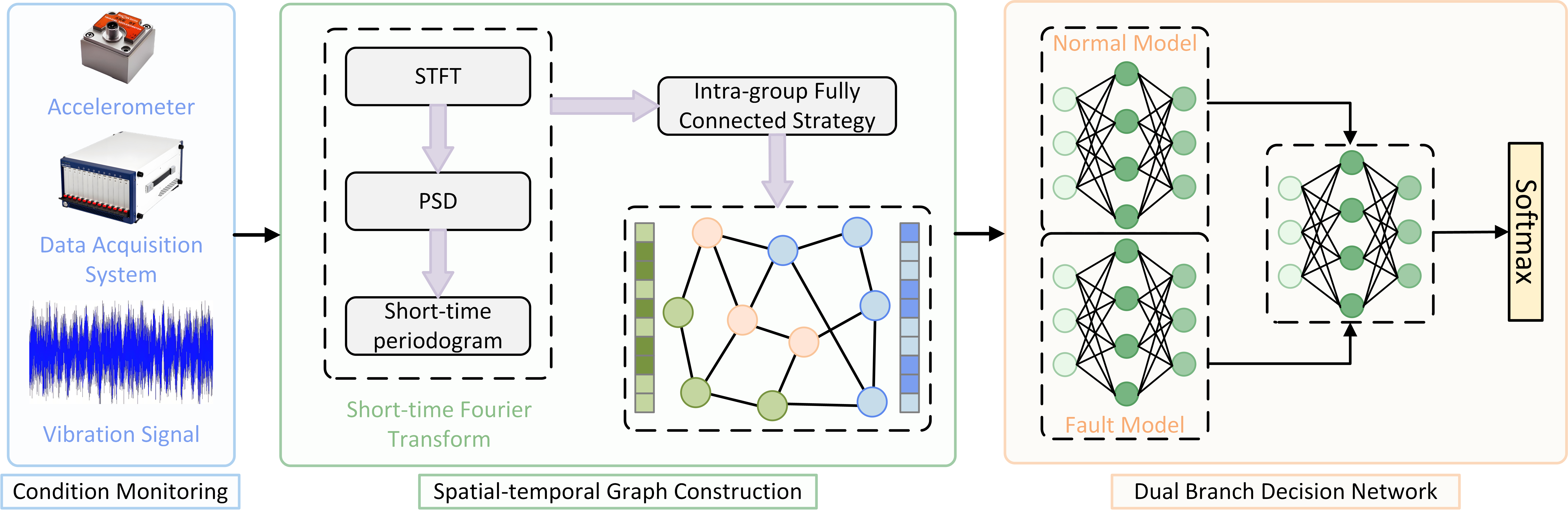

Supplement: Supplementary file 1 — Supplementary Material 1 [file 41598_2026_42504_MOESM1_ESM.zip › Supplementary/Architecture.png]

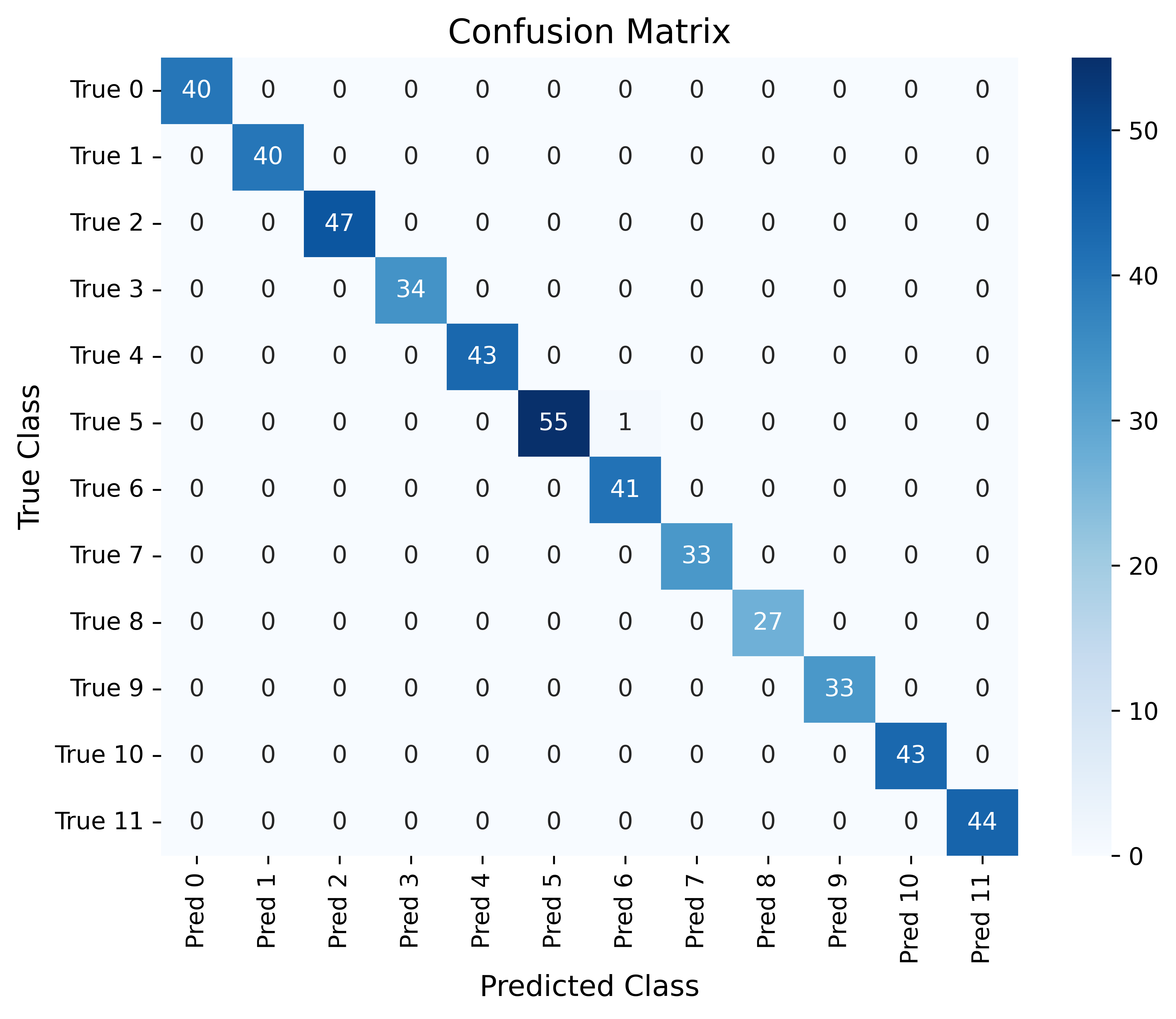

Supplement: Supplementary file 1 — Supplementary Material 1 [file 41598_2026_42504_MOESM1_ESM.zip › Supplementary/CWRU_confusion_matrix.png]

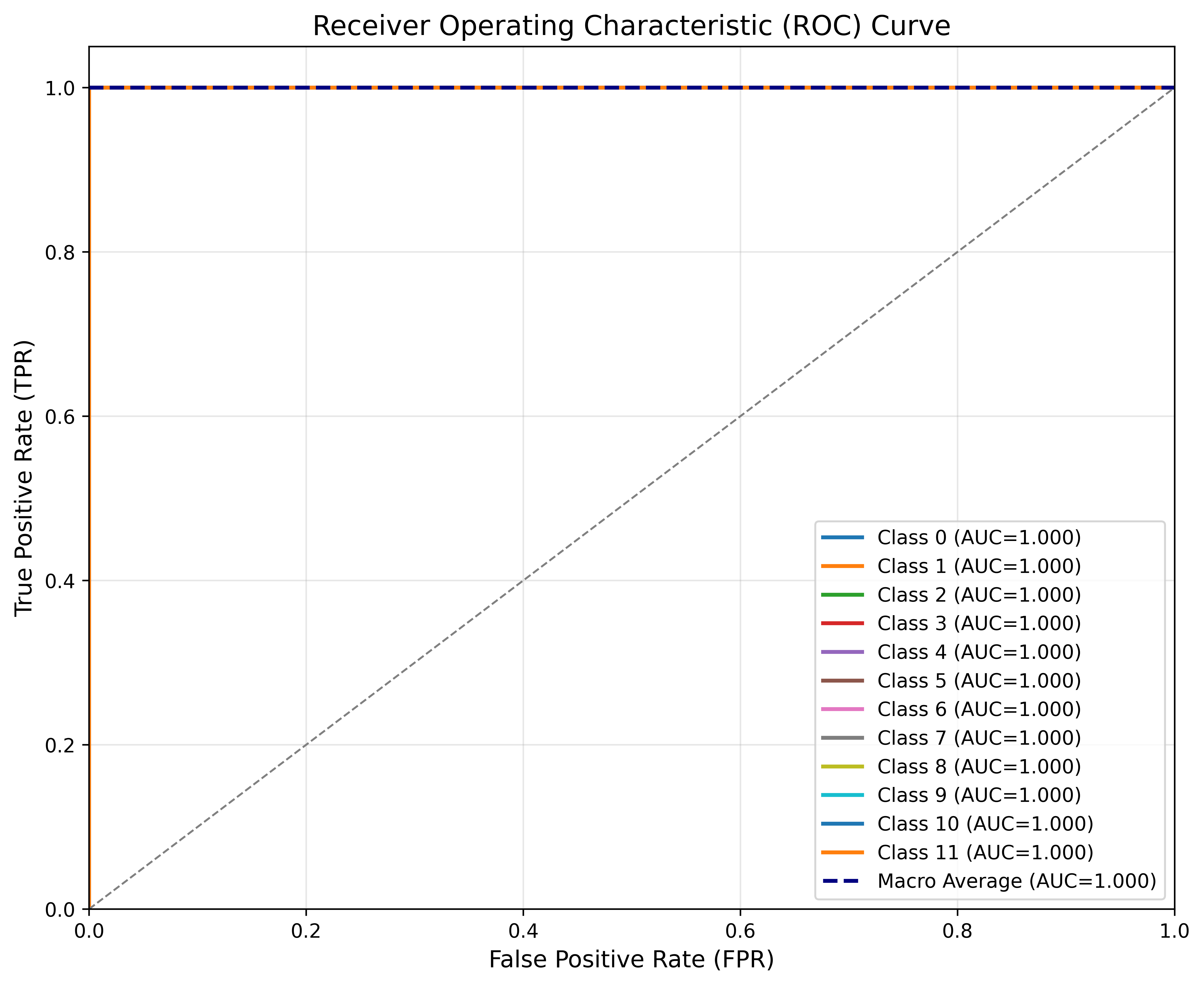

Supplement: Supplementary file 1 — Supplementary Material 1 [file 41598_2026_42504_MOESM1_ESM.zip › Supplementary/CWRU_roc_curve.png]

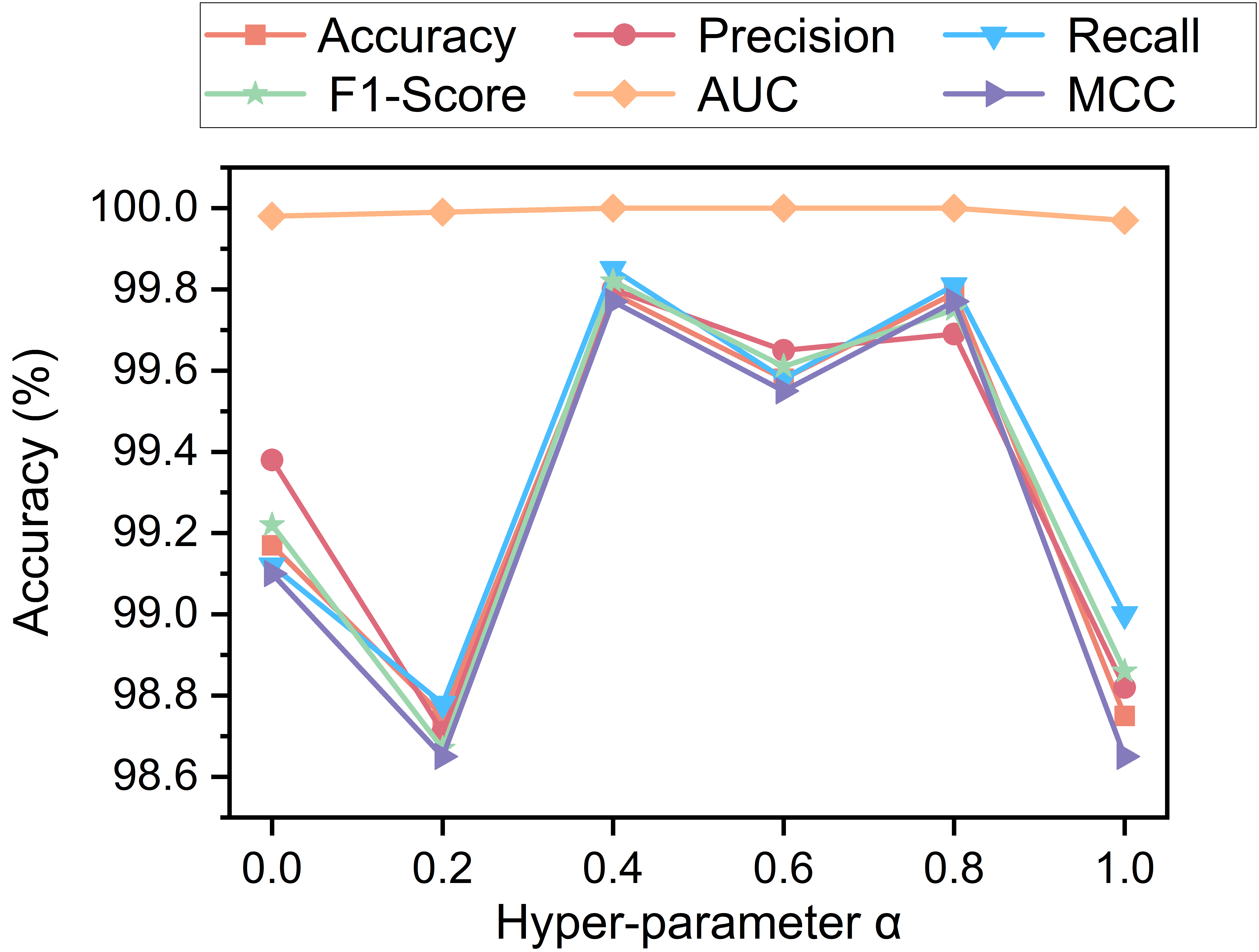

Supplement: Supplementary file 1 — Supplementary Material 1 [file 41598_2026_42504_MOESM1_ESM.zip › Supplementary/Parameter_sensitivity_CWRU.png]

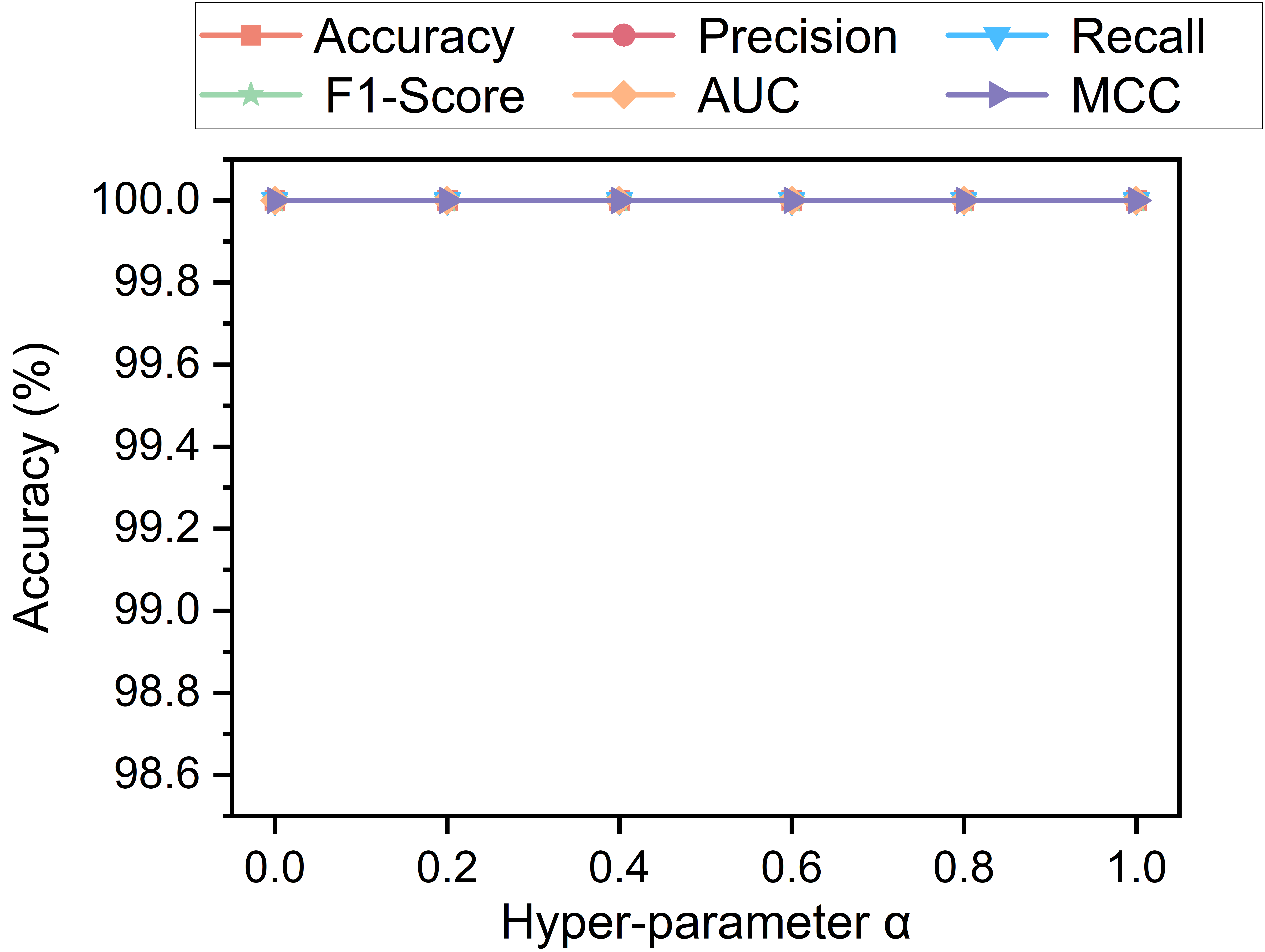

Supplement: Supplementary file 1 — Supplementary Material 1 [file 41598_2026_42504_MOESM1_ESM.zip › Supplementary/Parameter_sensitivity_PU.png]

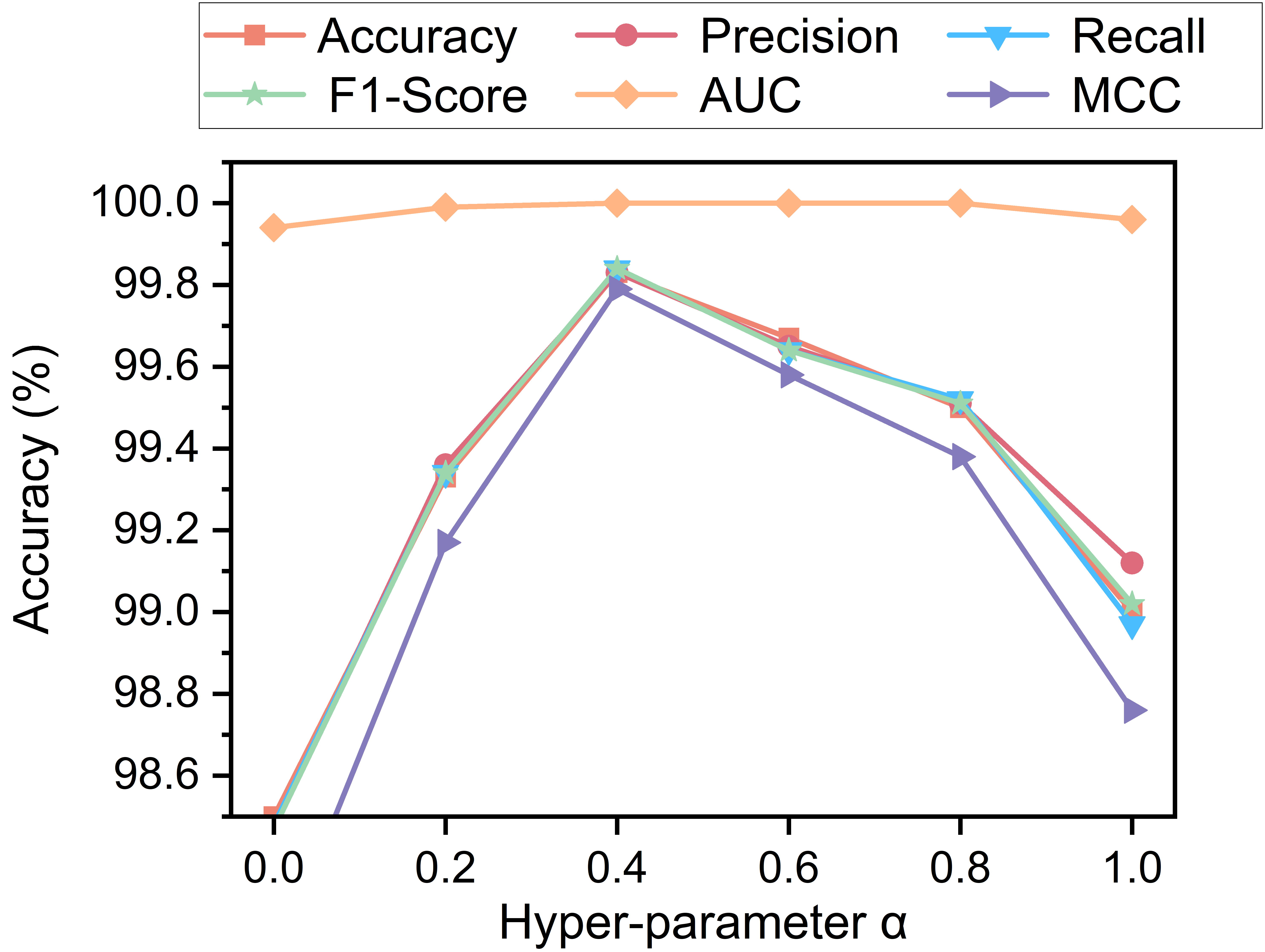

Supplement: Supplementary file 1 — Supplementary Material 1 [file 41598_2026_42504_MOESM1_ESM.zip › Supplementary/Parameter_sensitivity_uOttawa.png]

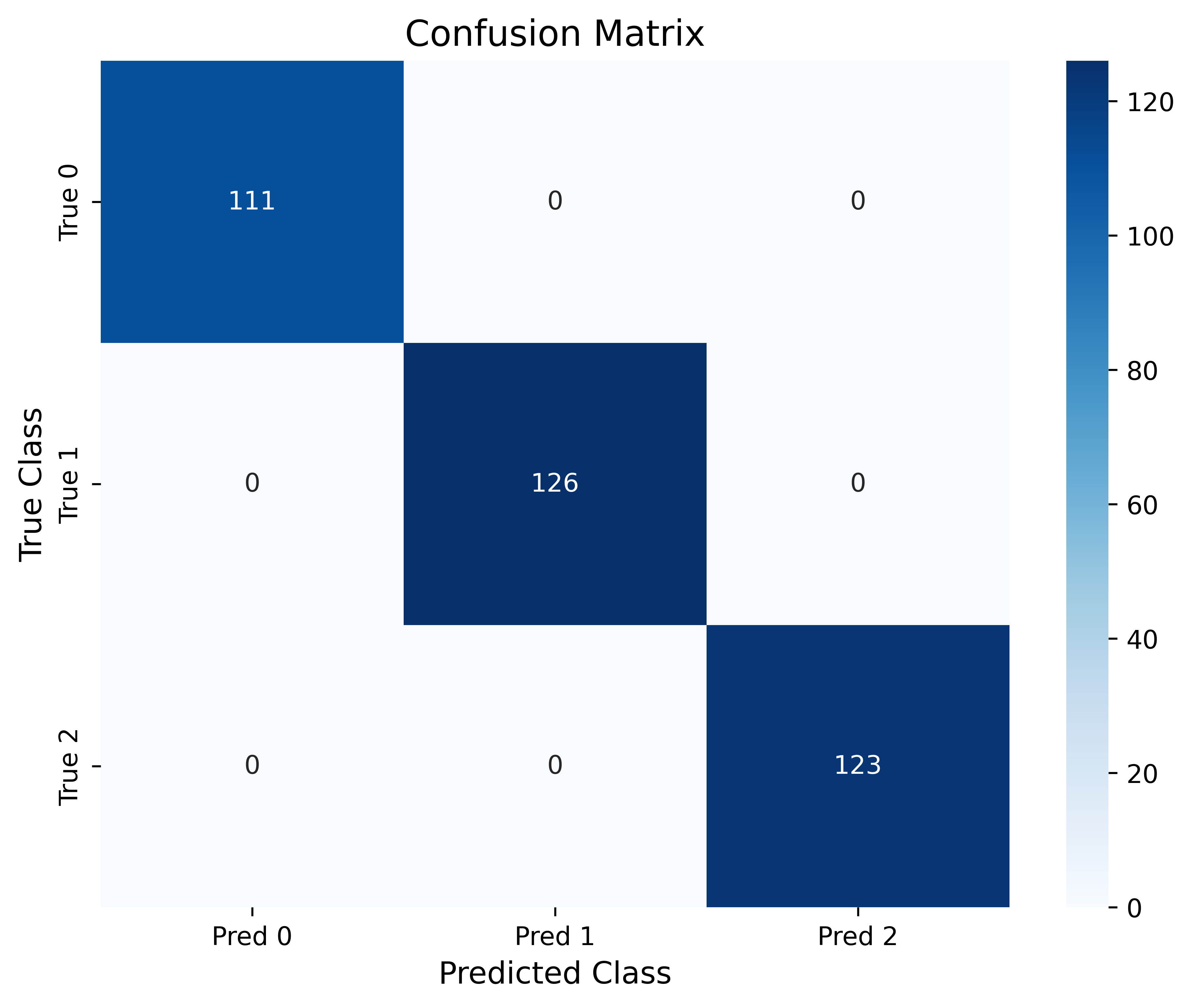

Supplement: Supplementary file 1 — Supplementary Material 1 [file 41598_2026_42504_MOESM1_ESM.zip › Supplementary/PU_confusion_matrix.png]

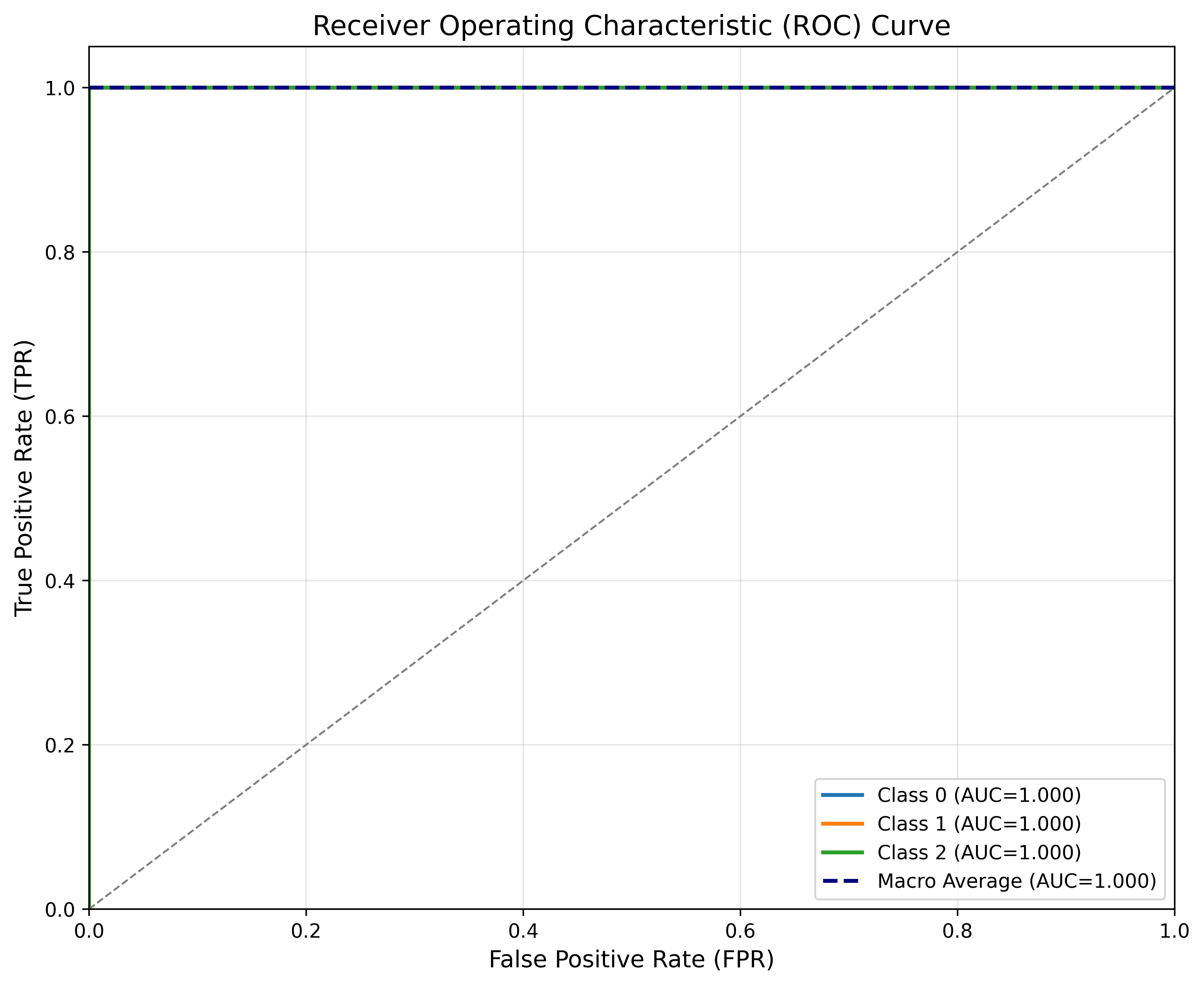

Supplement: Supplementary file 1 — Supplementary Material 1 [file 41598_2026_42504_MOESM1_ESM.zip › Supplementary/PU_roc_curve.png]

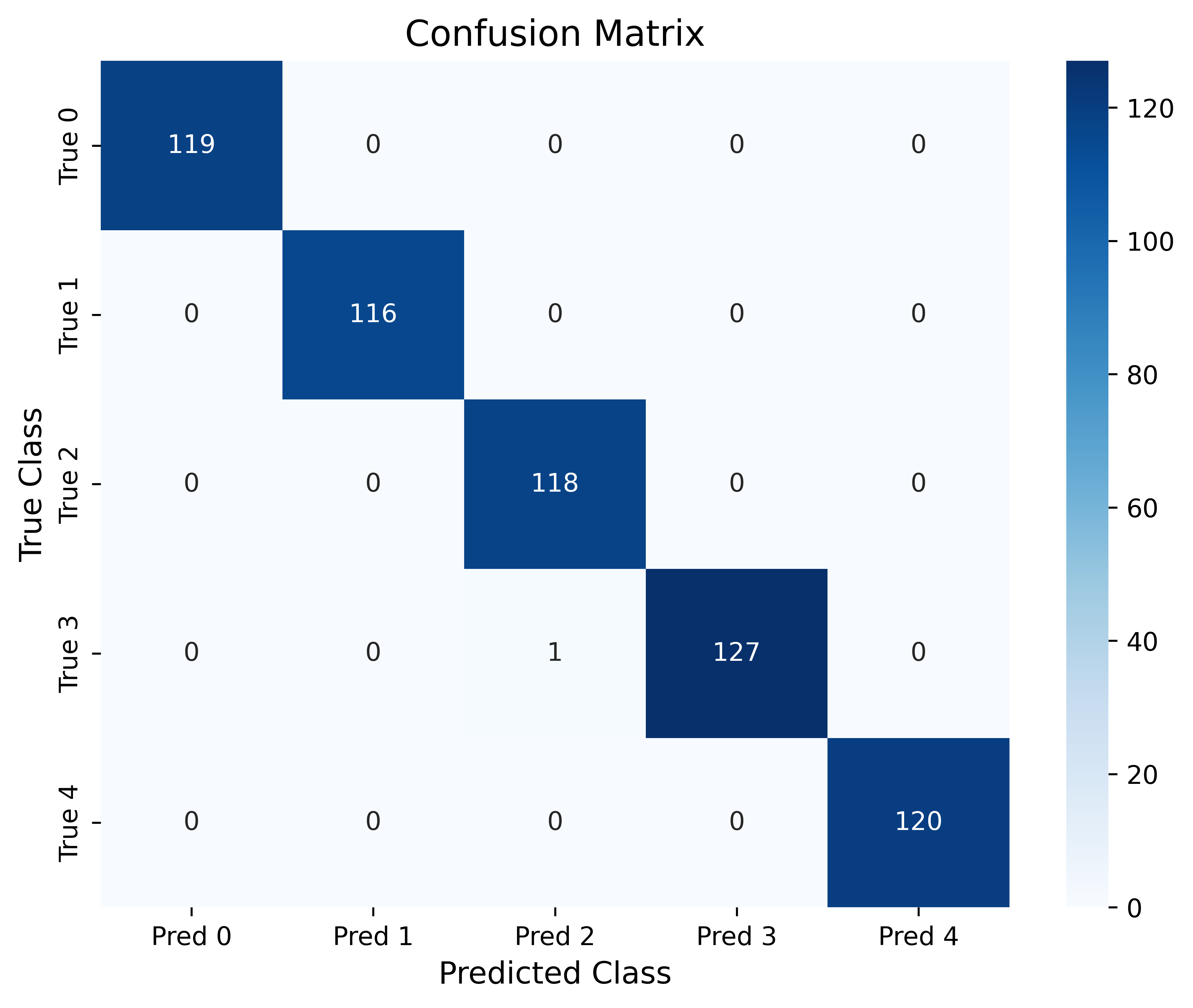

Supplement: Supplementary file 1 — Supplementary Material 1 [file 41598_2026_42504_MOESM1_ESM.zip › Supplementary/uOttawa_confusion_matrix.png]

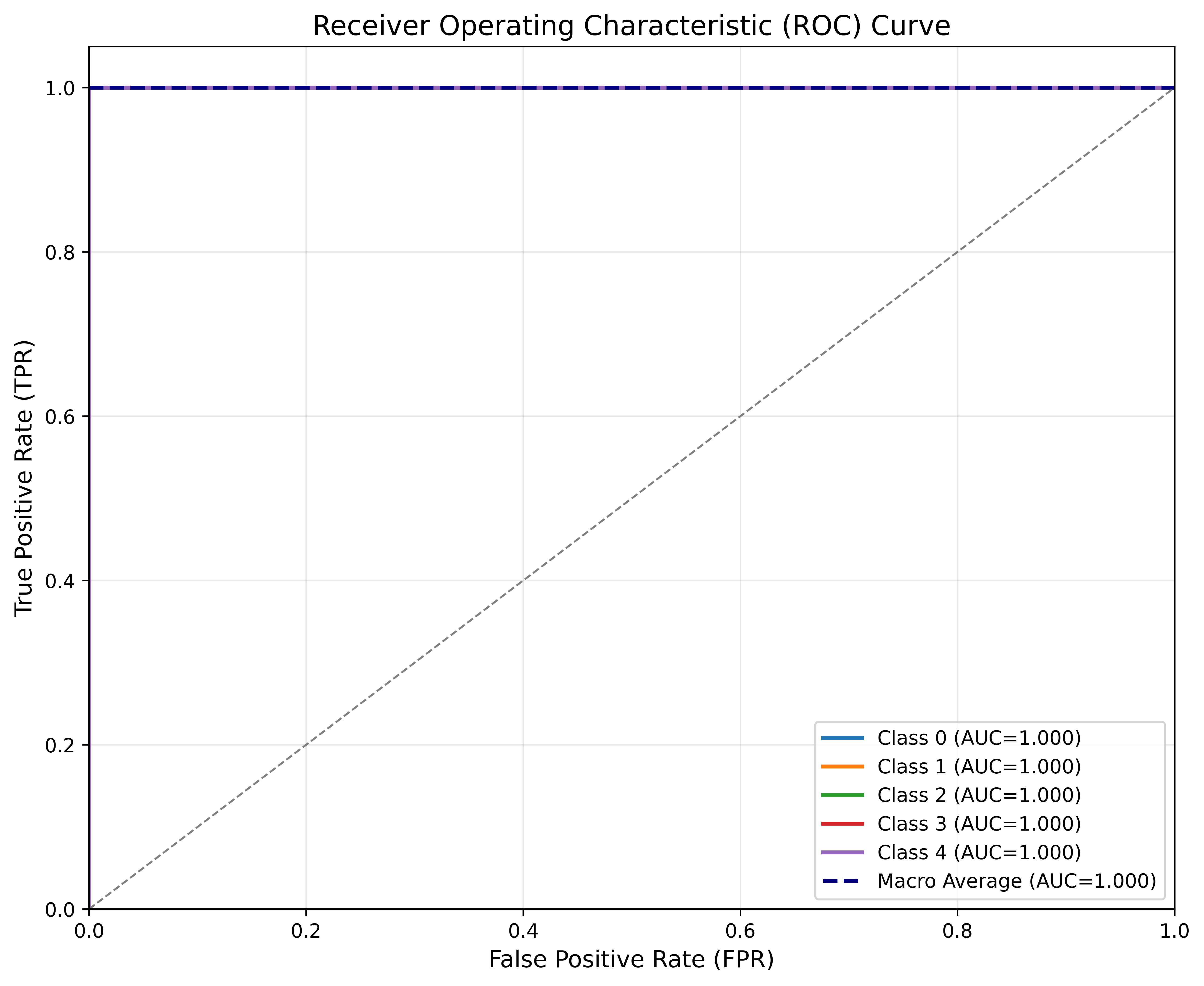

Supplement: Supplementary file 1 — Supplementary Material 1 [file 41598_2026_42504_MOESM1_ESM.zip › Supplementary/uOttawa_roc_curve.png]
